# 2024 Guidelines on Patient Blood Management for Adult Cardiac Surgery Under Cardiopulmonary Bypass in China

**DOI:** 10.31083/RCM31384

**Published:** 2025-06-16

**Authors:** Shujie Yan, Li Zhou, Chen Chen, Yuan Teng, Gang Liu, Luyu Bian, Jingjing Su, Hongwen Ji, Feilong Hei, Sheng Wang, Guyan Wang, Hushan Ao, Lei Du, Chengbin Zhou, Bingyang Ji, on behalf of the Working Group on CPB/ECLS of China NCCQI

**Affiliations:** ^1^Department of Cardiopulmonary Bypass, Fuwai Hospital, National Center for Cardiovascular Disease, Chinese Academy of Medical Sciences & Peking Union Medical College, National Clinical Research Center for Cardiovascular Diseases, 100037 Beijing, China; ^2^Department of Anesthesiology and Translational Neuroscience Center, West China Hospital, Sichuan University, 610041 Chengdu, Sichuan, China; ^3^Department of Cardiovascular Surgery, Guangdong Provincial Cardiovascular Institute, Guangdong Provincial People’s Hospital, Guangdong Academy of Medical Sciences, Southern Medical University, 510080 Guangzhou, Guangdong, China; ^4^Department of Cardiac Surgery, The First Affiliated Hospital of Harbin Medical University, 150001 Harbin, Heilongjiang, China; ^5^Department of Anesthesiology, Fuwai Hospital, National Center for Cardiovascular Disease, Chinese Academy of Medical Sciences & Peking Union Medical College, National Clinical Research Center for Cardiovascular Diseases, 100037 Beijing, China; ^6^Department of Cardiopulmonary Bypass, Beijing Anzhen Hospital, Capital Medical University, 100029 Beijing, China; ^7^Department of Anesthesiology, Beijing Anzhen Hospital, Capital Medical University, 100029 Beijing, China; ^8^Department of Anesthesiology, Beijing Tongren Hospital, Capital Medical University, 100005 Beijing, China

**Keywords:** guidelines, patient blood management, cardiac surgery, cardiopulmonary bypass

## Abstract

The Working Group on Cardiopulmonary Bypass and Extracorporeal Life Support of China National Center for Cardiovascular Quality Improvement presents evidence-based guidelines on patient blood management for adult cardiac surgery under cardiopulmonary bypass. These guidelines aim to promote patient blood management programs, reduce blood loss and allogeneic transfusion, and improve patient outcomes. The Guidelines Panel includes multidisciplinary experts. Based on prior investigation and the patient, intervention, comparison, outcome (PICO) principles, thirteen questions from four aspects were selected, including priming and fluid management during cardiopulmonary bypass, anticoagulation and monitoring during cardiopulmonary bypass, peri-cardiopulmonary bypass blood product infusion, and autologous blood infusion. Systemic reviews of the thirteen questions were performed through literature research. Recommendations were generated using the Grading of Recommendations Assessment, Development, and Evaluation (GRADE) system and reviewed and refined by all the Guideline Panel members. A total of 19 recommendations were finally approved after five expert meetings between 2023 and 2024. By implementing these recommendations, perfusionists and medical staff involved in cardiac surgery can optimize patient blood management, effectively decrease allogeneic blood transfusion rates, minimize perioperative bleeding, and ultimately improve the prognosis in adult patients undergoing cardiac surgery with cardiopulmonary bypass.

## 1. Introduction

More than 200,000 patients undergo cardiac surgery annually in China [1]. Cardiac surgery patients are among the highest consumers of allogeneic blood transfusions in hospitals. According to data from the Chinese Cardiac Surgery Registry, perioperative allogeneic blood transfusion rates in isolated coronary artery bypass grafting and isolated mitral valve surgery in 2022 were 42.3% and 55.5%, respectively [1]. Allogeneic blood transfusion increases the risks of blood-borne transmitted infections and transfusion-related adverse reactions. Growing evidence has shown that allogeneic transfusion is associated with increased mortality and morbidity in cardiac surgery patients.

Cardiopulmonary bypass (CPB) is the leading cause of increased perioperative blood transfusion in cardiac surgery. Full anticoagulation is required for CPB. In addition, CPB induces hemodilution due to the priming volume of the circuit, activates blood components through contact with artificial surfaces, and causes blood cells mechanical damage via blood pumps. These result in a decreased level of hemoglobin (Hb), consumption of coagulation factors and platelets, and hyperfibrinolysis, thus increasing the risks of perioperative anemia, massive blood loss, and allogeneic transfusion [2].

Patient blood management (PBM) is a patient-centered, systematic, evidence-based approach to improving patient outcomes by managing and preserving the blood of patients while promoting patient safety and empowerment. Thus, implementing a comprehensive perioperative PBM program in cardiac surgery could reduce hemodilution, blood component activation, and blood cell damage, decrease postoperative anemia, infection risks, and cardiovascular complications, and ultimately improve the outcomes of patients.

The perioperative PBM program comprises interventions against the CPB-related pathophysiologic process. The China National Center for Cardiovascular Quality Improvement has included implementing the perioperative transfusion rate and the PBM program within quality metrics. For years, the Chinese Society of Cardiothoracic and Vascular Anesthesiology and other academic societies have promoted the perioperative PBM program in cardiac surgery patients.

Meanwhile, societies in the United States and Europe have endorsed PBM guidelines for cardiac surgery [3, 4, 5, 6, 7]. In 2018, the Chinese Society of Cardiothoracic and Vascular Anesthesiology released the Expert Consensus on PBM in cardiac surgery [8]. Subsequently, following the increasing number of centers in China implementing comprehensive PBM programs in cardiac surgery [9, 10] and growing evidence with high quality [11, 12, 13] published in recent years, the strategies and interventions have been updated; for example, the latest guidelines from the Association for the Advancement of Blood and Biotherapies (AABB) recommended a threshold for red blood cell transfusion of 7.5 g/dL [14].

In China, certified perfusionists have different specialties and diverse backgrounds; therefore, the understanding and implementation of PBM can vary greatly. According to a 2021 survey of 181 cardiac centers in China [15], large-volume centers (performing ≥2000 cardiac surgeries annually) demonstrated significantly lower intraoperative packed red blood cells (pRBC) transfusion rates compared to small-volume centers (performing <2000 cases/year) (45% (23.59%, 55.00%) vs. 70% (30%, 90%), *p* = 0.008). Furthermore, large-volume centers were likelier to adopt strictive transfusion strategies and implement a broader range of PBM interventions. To promote perioperative PBM program in adult cardiac surgery, reduce blood loss and allogeneic transfusion, and improve patient outcomes, the Working Group on Cardiopulmonary Bypass and Extracorporeal Life Support of China National Center for Cardiovascular Quality Improvement presents evidence-based guidelines on PBM for adult cardiac surgery under CPB. These guidelines provide recommendations for all clinicians working in the field of PBM in cardiac surgery, emphasizing the PBM related to CPB.

## 2. Methods

The Working Group on Cardiopulmonary Bypass and Extracorporeal Life Support of China National Center for Cardiovascular Quality Improvement commissioned and approved this work. Bingyang Ji, Lei Du, and Chengbin Zhou were selected as chairpersons to assemble and coordinate the Guidelines Panel of multidisciplinary experts, including perfusionists, anesthesiologists, cardiac surgeons, intensivists, and transfusion medicine specialists **(Supplementary Material A)**. The guidelines were prepared according to the World Health Organization (WHO) Handbook for Guideline Development (second edition) [16] and the Basic Methods and Procedures for Formulating/Revising Clinical Practice Guidelines issued by the Chinese Medical Association [17]. Based on the prior investigation, the Guidelines Panel developed thirteen key clinical questions from four aspects according to the population, intervention, comparison, and outcome (PICO) format. These four aspects consist of priming and fluid management and anticoagulation and monitoring during CPB, alongside peri-CPB blood product infusion and autologous blood infusion. A systemic review was conducted for each question using MEDLINE, Embase, China Biology Medicine disc, Wanfang Database, and China National Knowledge Infrastructure (see keywords for each question in the **Supplementary Material B**). The literature search included systematic reviews, meta-analyses, and interventional and observational studies published from January 2000. The literature search was conducted in December 2023, and a pragmatic update was conducted in August 2024. All the relevant articles in English or Chinese were included for systemic review. Nineteen recommendations were generated for the questions. According to the Grading of Recommendations Assessment, Development, and Evaluation (GRADE) approach [18], the quality of evidence was initially determined by study design, with randomized controlled trials (RCTs) starting as high-quality and observational studies as low-quality. This initial rating was then adjusted based on five factors that may downgrade evidence (risk of bias, inconsistency, indirectness, imprecision, and publication bias) and three factors that may upgrade evidence (large effect size, dose-response gradient, and plausible confounding). The final quality of evidence was categorized as high, moderate, low, or very low (Table 1). Recommendations were graded as “strong” or “conditional” after considering the quality of the evidence, risk-benefit balance, the assumptions about the relative importance of outcomes, cost-effectiveness, acceptability, feasibility, and the context of national policy in China. Strong recommendations were those for which the panel was highly confident that the recommended option favorably balanced the expected benefits and risks for most patients in clinical practice. Conditional recommendations were those for which the panel was less confident that the potential benefits outweighed the risks.

**Table 1.** **Grading of Recommendations, Assessment, Development, and Evaluation (GRADE)**.

Certainty	Meaning	Examples*
High	There is a high level of confidence that the true effect is close to the estimated effect	Randomized trials without serious limitations
		Well-performed observational studies with very large effects (or other qualifying factors)
Moderate	There is a moderate level of confidence in the effect estimate; true effect is probably close to the estimated effect	Randomized trials with serious limitations
		Well-performed observational studies yielding large effects
Low	The confidence in the effect estimate is limited; true effect may be substantially different from the estimated effect	Randomized trials with very serious limitations
		Observational studies without special strengths or important limitations
Very low	There is very little confidence in the effect estimate; true effect is probably substantially different from estimated effect	Randomized trials with very serious limitations and inconsistent results
		Observational studies with serious limitations
		Unsystematic clinical observations (e.g., case series or case reports)

*The examples are not comprehensive. See text for criteria to downgrade or upgrade the quality of evidence.

An agreement was reached through conference calls and face-to-face meetings. All the Guideline Panel members reviewed the proposed recommendations. Members without a relevant conflict of interest voted on each recommendation to achieve consensus using a modified Delphi technique. Members were encouraged to provide open-ended feedback on each statement. Each guidance statement required a 75% voting participation rate and at least 80% consensus for approval and inclusion in the guidelines. Recommendations that did not meet these thresholds were revised based on the feedback received and redistributed for further voting. All recommendations reached a consensus after four rounds of revisions and voting.

## 3. Priming and Fluid Management During CPB

### Question 1. Should the priming volume be reduced to decrease the perioperative allogeneic transfusions?

**Recommendation 1. A CPB circuit with reduced priming volume is recommended as an effective measure of patient blood management (strong recommendation, high certainty)**.

**Recommendation 2. Retrograde autologous priming (RAP) is suggested to reduce perioperative allogeneic blood transfusion in adult cardiac surgery (strong recommendation, moderate certainty)**.

The total priming volume of the CPB circuit determines the degree of hemodilution. The ratio of priming volume to the estimated patient blood volume or body surface area is positively correlated with the risk of allogeneic transfusion [19, 20, 21].

Multiple measures were used to modify the circuit to reduce the priming volume, including shortening the circuit’s length, vacuum-assisted venous drainage, using oxygenators with integrated arterial filters, and closed circuits [9, 10, 21, 22, 23, 24, 25, 26]. For example, the modified low-priming CPB circuit (FUWAI-SAVE) used in several centers in China reduces the priming volume by approximately 40% compared to traditional extracorporeal circulation systems [9, 22, 23]. Based on the published literature, circuits with reduced priming volume could significantly reduce perioperative bleeding, decrease blood transfusion, and improve patient outcomes [9, 10, 22, 23, 24]. Moreover, RAP represents another noteworthy technique for reducing priming volume. RAP refers to introducing the patient’s blood into the CPB circuit after cannulation and before the bypass is initiated to displace the prime solutions. An RCT in China demonstrated RAP benefits patients with small body surface area [27]. A meta-analysis including 12 small RCTs and 17 observational studies showed that RAP can significantly reduce perioperative blood product transfusions [28]. When applying RAP technology, blood is drained from the body into the circuit after arterial cannulation and before initiating bypass, which can lead to hypovolemia and a subsequent drop in blood pressure. Therefore, RAP should be avoided or used with caution in patients with hemodynamic instability or those at high risk of hypovolemia, and close attention must be paid to maintaining the patient’s circulatory stability to ensure surgical safety.

### Question 2. Which solutions should be selected as the optimal priming solution?

**Recommendation 3. Balanced crystalloid solutions are recommended as the priming solution (strong recommendation, high certainty)**.

**Recommendation 4. In patients with preoperative hypoalbuminemia, adding human serum albumin into the priming solution may be considered (conditional recommendation, low certainty)**.

**Recommendation 5. Hydroxyethyl starch solution is not recommended for priming (strong recommendation, high certainty). However, a gelatin solution may be considered (conditional recommendation, very low certainty)**.

Crystalloids and colloid solutions are the priming fluid choices for CPB. Crystalloids comprise 0.9% saline and balanced crystal solutions, while colloids include human albumin and synthetic solutions (such as hydroxyethyl starch and gelatin). Different centers have significantly different preferences for priming solutions [29].

0.9% saline may be associated with an increased need for blood transfusion compared to balanced crystalloid solutions in non-cardiovascular surgery patients [30]. A Cochrane review that included 18 RCTs found that perioperative use of normal saline might increase the risk of hyperchloremic acidosis compared to balanced crystal solutions [31]. Based on the above research, the balanced crystalloid solution is recommended for priming.

Numerous studies have compared the impact of different priming strategies of the crystalloid solution, human albumin, and artificial colloid on perioperative blood transfusion and postoperative bleeding. However, no unified conclusion has been reached. Meta-analyses with different inclusion criteria, publication times, comparison methods, and study endpoints also yielded inconsistent findings [32, 33, 34, 35, 36, 37].

Although clinical research in the field of critical care suggested that human albumin is associated with favorable outcomes [38, 39], and the evidence showed that albumin might have the potential role in protecting the endothelial glycocalyx of the endothelium [40], its effect in cardiac surgery under CPB remains unclear. A recent single-center, randomized, double-blind, controlled trial [41, 42] that included 1407 adult patients undergoing cardiac surgery under CPB compared the effects of 4% albumin solution and acetate Ringer’s solution used in the priming solution and perioperative fluid therapy on major postoperative complications. The study found that the albumin group had higher rates of bleeding events and perioperative blood product transfusions than the Ringer’s solution group, with no difference in the incidence of perioperative adverse events between the two groups. Influenced by this study, a recent network meta-analysis [34] showed that the albumin priming strategy might increase the risk of red blood cell transfusion compared with the crystalloid priming strategy. Another meta-analysis [35] showed that albumin infusion during cardiac surgery did not reduce perioperative mortality. Based on the above studies and the significantly higher cost of human albumin compared to other solutions, it is not recommended to use albumin in CPB priming solutions routinely. However, for patients with preoperative hypoalbuminemia, the use of albumin in priming may be beneficial owing to evidence that perioperative hypoalbuminemia is independently associated with poor prognosis in cardiac surgery patients [43, 44].

A hydroxyethyl starch-based solution is a nonionic starch derivative, which has been proven to be associated with the risk of perioperative bleeding, coagulation impairment, increased blood product transfusion, and renal injury by several studies and meta-analyses [45, 46, 47, 48, 49]. Based on the above risks, the China National Medical Products Administration issued an announcement in 2022 regarding revisions of the instructions for hydroxyethyl starch injections [50], which recommended against the use of hydroxyethyl starch in cardiac surgery with CPB. The United States and Europe have also issued warning statements for the clinical use of hydroxyethyl starch [51].

Gelatin solutions are colloid solutions of animal origin. After the warning against hydroxyethyl starch solutions, gelatin is currently the most commonly used synthetic colloid for priming fluids in China. However, the limited studies conducted on gelatin presented small sample sizes and low-quality data. These studies did not show an increased risk of bleeding, kidney injury, or prolonged intensive care unit (ICU) time [52]. Thus, it is important to be cautious when using gelatin solutions, as they can easily cause allergic reactions [53], mostly manifesting as a decrease in blood pressure.

In summary, the weight of colloids in CPB fluid therapy has shifted downward due to an understanding of the structure of endothelioglycalyx, the revised Starling’s Principle, and the reduction in priming volume. Recent studies have found that albumin increases the risk of blood transfusion, driving us to rethink whether colloid is needed in the priming solutions [34].

### Question 3. Should ultrafiltration be used during CPB to reduce allogeneic transfusion?

**Recommendation 6. For patients with large priming volumes, large blood volumes, or anticipated large residual pump blood volumes, ultrafiltration should be considered part of PBM (strong recommendation, moderate certainty)**.

Ultrafiltration consists of conventional ultrafiltration (CUF), zero-balance ultrafiltration, and modified ultrafiltration (MUF). A meta-analysis [54] that included 10 RCTs and 1004 patients showed that ultrafiltration could reduce perioperative blood transfusion and postoperative bleeding in adult cardiac surgery. However, the study did not analyze patients according to different forms of ultrafiltration. Another meta-analysis in 2024 [55] examined CUF (two observational studies, n = 47,007) and MUF (seven RCTs, n = 928; the sample size of the largest study was n = 573 [56]) separately and showed that modified ultrafiltration reduced intraoperative RBC transfusion, whereas conventional ultrafiltration did not. The two observational studies included in this meta-analysis were multicenter retrospective studies [57, 58]. Differences in transfusion thresholds among multiple centers might affect the evaluation of the blood-conservation effect of CUF. MUF was previously widely used in small-weight children; however, the technique has recently been gradually expanded to adult patients. Another meta-analysis [59] also demonstrated that modified ultrafiltration in adult cardiac surgery could increase postoperative hemoglobin levels, decrease postoperative drainage, and reduce blood transfusions. However, the above studies were published early, and the priming volumes were relatively large (1500–2000 mL). Hence, the value of ultrafiltration as a PBM measure in low-priming CPB requires further study. For patients with large priming volumes, large blood volumes, or those expected to have large volumes of residual pump blood, ultrafiltration should be considered a PBM measure. In addition, it should be noted that several studies [57, 60, 61] have shown that excessive ultrafiltration volume during CPB might increase the risk of postoperative acute kidney injury (AKI). A multicenter registry [57] study further highlighted that a patient’s preoperative renal function influences the risk of AKI. Specifically, among patients with a creatinine clearance of less than 99.6 mL/min, a higher volume of CUF was associated with an elevated risk of AKI. Therefore, in patients with preoperative renal dysfunction, the volume of CUF should be carefully controlled to avoid excessive use. The Guideline Panel recommends the reduced priming volume strategy to lower perioperative fluids infusion rather than excessive ultrafiltration.

It is worth noting that fluid management, including priming volume, fluid selection, ultrafiltration application, or fluid balance management, should be individualized for each patient. This represents an important direction for future research.

## 4. Anticoagulation and Monitoring During CPB

### Question 4. How should heparization and protamine neutralization be managed appropriately?

**Recommendation 7. Fresh frozen plasma or antithrombin Ⅲ (ATⅢ) concentrate could be considered in patients with heparin resistance who could not achieve an activated clotting time (ACT) target of 480 seconds after the administration of unfractionated heparin 600 U/kg (conditional recommendation, moderate certainty)**.

**Recommendation 8. Continuous monitoring of heparin residual and rebound may be considered from the initial protamine neutralization to 6 h after surgery (conditional recommendation, moderate certainty)**.

Unfractionated heparin (UFH) is the standard anticoagulant for CPB. UFH produces its major anticoagulant effect by binding its pentose sequence to ATⅢ and enhancing the inhibition of ATⅢ on coagulation factors [62]. Heparin resistance refers to the inability to achieve the targeted anticoagulation levels with sufficient UFH doses. Currently, there is no universal definition of heparin resistance for CPB. In 2024, a review by the Scientific Standards Committee of the International Society for Thrombosis and Hemostasis [63] proposed a standardized definition for heparin resistance as the inability to obtain an ACT target for CPB of 480 seconds or more after 500 U/kg. The primary approach to managing heparin resistance remains increasing the heparin dose; however, the maximum dose of UFH administered before initiating ATⅢ supplementary therapy to achieve a target ACT varied among different centers. According to a 2019 survey, the majority of the respondents selected 600 U/kg as the preferred threshold [64]. ATⅢ could be supplemented by infusing fresh frozen plasma or administering ATⅢ concentrates [65, 66, 67]. Each milliliter of fresh frozen plasma contains 1 International Unit (IU) ATⅢ, meaning 5–15 mL/kg of fresh frozen plasma could be adequate to correct heparin resistance; the suggested dose of ATⅢ concentrates is 500–1000 IU. Despite the lack of published clinical trials directly comparing fresh frozen plasma and AT concentrates, some literature generally favored ATⅢ as a safe, efficient, and effective choice for the clinical management of heparin resistance; its benefits include lower volume administration, less risk of transfusion-related acute lung injury and lower risk of transfusion-related infections, as well as avoiding volume overload and intraoperative time delay. However, antithrombin concentrate may pose a risk of heparin rebound in patients in the early postoperative period [65, 66], and the price of ATⅢ concentrates affects the commercial application in China. The Guideline Panel suggested that when the ACT value could not reach 480 s after administering 600 U/kg UFH before CPB, the fresh frozen plasma or ATⅢ concentrates could be considered.

After weaning from CPB, protamine is administered to neutralize the effect of UFH. Protamine binds its cationic arginine group to anionic heparin in a 1:1 ratio through electrostatic binding, forming a salt complex cleared by the reticuloendothelial system [68]. Inadequate protamine could leave residual-free heparin in the blood, but excess protamine could impair coagulation function. The impact of protamine overdose on coagulation may be attributed to several mechanisms, including effects on platelet function, inhibition of the Glycoprotein Ib-von Willebrand Factor interaction, reduced thrombin generation, and impaired activation of clotting factors such as factor V, factor VII, and factor VIII. Meanwhile, the optimal strategy for protamine dosing is controversial due to the difficulty in estimating heparin concentrations *in vivo*; the conventional method is to administer protamine at a ratio of about 1:1 according to the initial or total heparin dose. However, a study [69] found that an initial heparin dose-based 1:1 protamine dosing strategy might lead to protamine overdosing by inhibiting the coagulation process. In comparison, an individualized protamine dosing strategy is more appropriate. Currently, several titration methods have been proposed, including the hemostatic management system (HMS) titration strategy [70, 71, 72], ACT-based model dosing strategy [73], statistical model dosing strategy [74], and pharmacokinetic model dosing strategy [75]. However, these titration methods are only supported by small studies involving patients with a low risk of bleeding [76]. Thus, more research is needed to find a more accurate and cost-effective method for measuring heparin concentration or develop a better model to calculate heparin concentrations in patients with a high risk of bleeding [77].

Heparin rebound occurs when detectable heparin blood levels are present remotely after adequate heparin reversal with protamine. One study suggested that 10% to 15% of patients receiving usual heparin doses for CPB would have detectable heparin levels 2 hours after protamine reversal [78]. A small prospective study [79] measured plasma heparin concentrations at 1 hour, 2 hours, 4 hours, 6 hours, and 24 hours following the end of the protamine infusion and found residual heparin appeared in all patients 2 hours later, with the peak heparin concentration occurring at the 4-hour time-point. A randomized trial involving 300 patients showed that a continuous protamine infusion after initial protamine reversal (25 mg/h for 6 hours) could abolish heparin rebound and result in modest but significant reductions in chest tube drainage but not transfusion requirements [80]. Based on the above evidence, the Guideline Panel suggested continuous monitoring of heparin rebound within 6 hours postoperatively either by HMS, ACT, or viscoelastic testing to guide protamine redosing. Specifically, we suggested monitoring for heparin rebound at 2-hour, 4-hour, and 6-hour following cardiac surgery.

UFH is contraindicated for patients with acute or subacute heparin-induced thrombocytopenia who are awaiting emergency cardiac surgery under CPB, and bivalirudin is the first-line alternative. In cases where HIT is complicated by severe renal insufficiency, argatroban is preferred due to its non-renal clearance pathway [81].

### Question 5. How should anticoagulation be appropriately monitored during CPB?

**Recommendation 9. Maintaining ACT above 480 seconds (celite method) during CPB is recommended (strong recommendation, moderate certainty). It is reasonable to determine the appropriate target ACT values of different instruments for CPB based on the instructions (conditional recommendation, low certainty)**.

**Recommendation 10. The ACT value should be monitored regularly during CPB (every 30–60 minutes) to guide heparin redosing (strong recommendation, low certainty)**.

ACT is the gold standard for anticoagulation monitoring in CPB. The establishment of the ACT threshold for CPB is based on the earlier observations of clot or fibrin formation in the circuits of CPB. An ACT above 480 seconds was believed to be a threshold with reasonable safety and is still in use. Although the evidence is insufficient, the threshold of 480 seconds has been written into the CPB standards, guidelines, and textbooks [68, 82]. The half-life of 400 U/kg heparin is 152 ± 5 min [83]. Therefore, monitoring the ACT value regularly every 30 to 60 minutes during CPB to guide heparin redosing is reasonable. Moreover, shortening the ACT monitoring interval for patients with preoperative low plasma ATⅢ levels, resistance to heparin, and a large amount of ultrafiltration during CPB or large urine volume is likewise reasonable.

Presently, multiple commercial instruments can automatically detect the ACT values. Celite was the activator of early ACT instruments, while current ACT monitoring equipment may also use activators such as kaolin and phospholipids. Thus, the ACT values detected by these devices are slightly different due to the difference in activators [84, 85]. ACT equipment using a celite activator tends to provide higher values at lower ACT ranges while returning lower values at the higher ACT range when compared to the kaolin activator [84]. Even with different ACT devices using the same activator, ACT measurement values may still deviate [86]. Hence, it is reasonable to determine the appropriate ACT thresholds for CPB for different ACT devices based on ACT bias values with the traditional celite-based instrument.

### Question 6. Should viscoelastic testing be performed to guide hemostasis management and reduce bleeding?

**Recommendation 11. For patients with coagulopathy, thromboelastogram (TEG)/rotational thromboelastometry (ROTEM) is recommended to guide hemostasis management and blood product transfusion after weaning off CPB (strong recommendation, high certainty)**.

Viscoelastic point-of-care (POC) whole blood tests, including TEG and rotational ROTEM, are commonly used for diagnosing perioperative coagulation function in cardiac surgery [87, 88]. TEG and ROTEM have similar parameters [89], reflecting the entire process from blood coagulation to fibrinolysis. Multiple studies [90, 91, 92, 93, 94, 95] have shown that TEG/ROTEM monitoring during on-pump cardiac surgery could not only reduce blood product infusion and bleeding but might also lower the risks of postoperative AKI [94] and mortality [95]. The multicenter RCT with the largest sample size (n = 7402) showed that the TEG/ROTEM-based transfusion algorithm started from CPB rewarming could reduce allogeneic transfusions and major bleeding following cardiac surgery [91]. TEG/ROTEM has been recommended for perioperative hemostasis management by multiple guidelines and expert consensus [6, 96, 97]. Some centers have developed institutional TEG/ ROTEM-based transfusion algorithms during and after CPB [90, 91, 95] (See examples for TEG/ROTEM-based hemostatic interventions and transfusion algorithms in **Supplementary Material C**). However, the optimal monitoring timing and approach still need further study.

### Question 7. Should antifibrinolytic therapy be used to reduce bleeding and transfusion?

**Recommendation 12. The prophylactic administration of tranexamic acid is recommended to reduce bleeding and blood product transfusion (strong recommendation, high certainty)**.

Surgical procedures and CPB cause hyperfibrinolysis, leading to perioperative bleeding. Tranexamic acid is a synthetic lysine analog that inhibits fibrinolysis by competitively inhibiting plasmin and plasminogen and is regarded as a mainstay antifibrinolytic agent in cardiac surgery due to its safety and high antifibrinolytic efficacy [98]. Previous studies [99, 100, 101] have shown that tranexamic acid reduced bleeding and blood transfusion without increasing postoperative deep vein thrombotic complications or death. However, the dosage of tranexamic acid has been debated, and the effects of different doses of tranexamic acid on patient outcomes have been studied [13, 102, 103, 104]. Although high doses of tranexamic acid are more likely to reduce blood loss and infection, a high dosage might increase the risk of seizure [103]. In 2022, a multicenter RCT (optimal study) [13] involving 3079 Chinese adult patients undergoing cardiac surgery with CPB showed that a high dosage (a 30 mg/kg bolus, a 16 mg/kg/h maintenance dose, and a 2 mg/kg prime) compared with a low dose (a 10 mg/kg bolus, a 2 mg/kg/h maintenance dose, and a 1 mg/kg prime) of tranexamic acid infusion resulted in a modest statistically significant reduction in the proportion of patients who received allogeneic red blood cell transfusion and met criteria for noninferiority concerning a composite primary safety endpoint.

Epsilon aminocaproic acid is also a lysine analog. Several studies [105, 106, 107] compared the effectiveness of epsilon aminocaproic acid to tranexamic acid in reducing blood loss and transfusion requirements in patients undergoing cardiac surgery. These studies found no significant difference between the two drugs. However, there is limited evidence on epsilon aminocaproic acid, and further large-scale studies are needed to demonstrate its efficacy and safety.

## 5. Peri-CPB Blood Product Infusion

### Question 8. Should a restrictive transfusion strategy be applied in cardiac surgery?

**Recommendation 13**. **A restrictive transfusion strategy is recommended (strong recommendation, high certainty). Generally, it is suggested that Hb levels ≥7 g/dL should be maintained during CPB and that Hb ≥8 g/dL should be maintained after CPB (strong recommendation, moderate certainty). An individualized Hb target strategy based on oxygen delivery–consumption balance may be considered (conditional recommendation, moderate certainty)**.

During CPB, patients’ Hb levels tend to decrease due to hemodilution, hemolysis, and blood loss. Evidence from recent RCTs, systematic reviews, and meta-analyses supports that a restrictive transfusion strategy during the perioperative period of cardiac surgery can reduce perioperative red blood cell transfusion requirements without increasing the risk of mortality or major adverse clinical events. The TRICS III study [11, 12], a multicenter RCT that included 5243 adult patients undergoing on-pump cardiac surgery with a EuroSCORE of ≥6, found that a restrictive transfusion strategy (transfusing red blood cells only when Hb <7.5 g/dL) significantly reduced transfusion rates compared to a liberal strategy (transfusing when Hb <9.5 g/dL in the operating room or ICU, or Hb <8.5 g/dL on the ward) without increasing the risk of adverse outcomes at discharge or six months follow-up. Based on the TRICS III study, the 2023 AABB red blood cell transfusion guidelines [14] and the 2019 guidelines from the Society of Cardiovascular Anesthesiologists [108] recommend a perioperative transfusion threshold of 7.5 g/dL in cardiac surgery.

Hypothermia and anesthesia during CPB reduce oxygen consumption, meaning the transfusion threshold during CPB could be slightly lower than 7.5 g/dL. A large retrospective study in China [9] demonstrated that maintaining a target Hb level of ≥7 g/dL during CPB is safe. However, the optimal postoperative Hb threshold remains debated. A dual-center retrospective study also in China that included 8206 patients [109] reported a significant increase in in-hospital mortality risk when the postoperative Hb fell below 7 g/dL. The commonly adopted postoperative transfusion threshold of 8 g/dL in major Chinese centers [8, 9] has shown favorable patient outcomes. Therefore, based on the above evidence, this guideline recommends maintaining a target Hb level of ≥8 g/dL post-CPB.

Moreover, a fixed Hb threshold may not be suitable for all cases; patient factors such as gender, age, comorbidities (e.g., older patients, heart failure, hypoxemic), temperature, oxygen delivery–consumption balance, cardiac and pulmonary functions, and CPB flow should also be considered. The Hb threshold for transfusion should be individualized. Furthermore, strictly adhering to a restrictive transfusion strategy without considering the patient’s clinical condition may increase the risk of tissue ischemia, inadequate oxygen delivery, postoperative delirium, and other adverse events. There is a growing consensus that the risks and benefits of restrictive versus liberal transfusion strategies may vary from person to person. Intraoperative monitoring tools that reflect oxygen delivery–consumption balance, such as blood lactate concentration, cerebral tissue oxygenation, and venous oxygen saturation, can help inform individualized decisions. For instance, a retrospective study [110] indicated that red blood cell transfusions during CPB are beneficial when venous oxygen saturation (SvO_2_) drops below 68% and/or the oxygen extraction ratio exceeds 39%. Additionally, multiple guidelines [111] recommend goal-directed perfusion based on oxygen delivery (DO_2_), suggesting that Hb targets should be individualized according to DO_2_ and achievable CPB flow or cardiac output.

It is important to emphasize that preoperative anemia management is critical in reducing intraoperative transfusion requirements and ensuring adequate Hb levels and oxygen supply during surgery. For patients with preoperative anemia, intravenous ferric carboxymaltose (1000–1500 mg) within 4 weeks in patients with preoperative iron deficiency or non-anemic iron deficiency may reduce RBC transfusion, or erythropoiesis-stimulating agents (40,000 IU weekly combination intravenous iron) in patients with refractory anemia may reduce transfusion rates [112, 113, 114].

### Question 9. How should other blood products be used appropriately?

**Recommendation 14. Prothrombin complex concentrate (PCC) may be used for patients with severe bleeding after CPB due to the confirmed deficiency of clotting factors (conditional recommendation, low certainty)**.

**Recommendation 15. For patients with severe bleeding and low fibrinogen levels after CPB, fibrinogen supplementation may be considered as it is not inferior to cryoprecipitate in efficacy (strong recommendation, moderate certainty)**.

PCC contains concentrated vitamin K-dependent factors (II, VII, IX, X). Fresh frozen plasma (FFP) is reserved for multifactorial coagulopathy (e.g., combined factor deficiencies without a dominant deficit). Routine transfusion of FFP and PCC is not generally recommended in cardiac surgery under CPB unless there is significant post-CPB bleeding or a confirmed deficiency of clotting factors. Retrospective studies on the relationship between post-CPB FFP transfusion and patient outcomes have yielded conflicting results [115, 116]. One study [115] (n = 12,043) found that FFP transfusion did not increase the risk of in-hospital mortality, infection, or acute kidney injury. In contrast, another study [116] (n = 119,138) reported an association between FFP transfusion and an elevated 30-day postoperative mortality risk. The PROPHESY study [117] (n = 50) observed no statistically significant difference in clotting factor levels after FFP or PCC infusion in patients with post-CPB bleeding. However, a meta-analysis [118] that included eight studies involving 1500 cardiovascular surgery patients found that PCC transfusion significantly reduced chest tube drainage and transfusion requirements within 24 hours postoperatively. A meta-analysis estimated that PCC could indirectly reduce hospitalization costs through approximately 10% decrease in the RBC transfusion [119]. Therefore, in cases of severe bleeding and clotting factor deficiency post-CPB, PCC transfusion may be considered.

Both cryoprecipitate and human fibrinogen concentrate can treat fibrinogen deficiency following CPB. In addition to fibrinogen, cryoprecipitate provides VIII, XIII, and von Willebrand factors. A cryoprecipitate dose of 1 unit per 10 kg body weight (approximately 15 mL) can increase plasma fibrinogen levels by 0.5 g/L [120]. Currently, only one small prospective study [121] (n = 13) has evaluated the use of cryoprecipitate in CPB cardiovascular surgery, finding that cryoprecipitate administration improved fibrinogen levels and fibrin-based clot strength in patients undergoing deep hypothermic circulatory arrest during aortic surgery. Another RCT [122] (n = 48) found that preoperative supplementation with human fibrinogen concentrate in coronary artery bypass graft patients increased intraoperative and postoperative fibrinogen levels but did not reduce postoperative bleeding. Similar findings were reported in an RCT by Bilecen *et al*. [123]. Another study [124] also observed an increased risk of allogeneic blood product transfusion in patients receiving fibrinogen supplementation during aortic surgery. Consequently, routine prophylactic use of human fibrinogen to reduce postoperative bleeding and transfusion requirements is not recommended. However, for patients with confirmed hypofibrinogenemia, human fibrinogen concentrate is as effective as cryoprecipitate for fibrinogen supplementation [125, 126], with similar thromboembolic events reported in both groups [127]. Given its ease of administration, high purity, cost-effectiveness, and strong efficacy in raising fibrinogen levels, human fibrinogen concentrate may be preferred over cryoprecipitate in patients with hypofibrinogenemia following CPB.

Currently, large-scale studies on perioperative platelet transfusion in cardiovascular surgery are lacking. One retrospective study [128] involving 12,043 cardiovascular surgery patients found that platelet transfusion was associated with reduced infection risk and shorter ICU and hospital stays. Another retrospective study [129], which included 119,132 patients from 40 centers, observed an association between platelet transfusion and lower in-hospital and 90-day mortality and reduced risks of deep wound infection and acute kidney injury. However, a separate study [130] indicated that in patients experiencing massive bleeding post-CPB, sequential transfusion of four units of single-donor platelets increased platelet count but did not improve platelet aggregation function or hemostasis.

Therefore, more high-quality research is needed to provide stronger evidence on the relationship between perioperative platelet transfusion and outcomes regarding hemostasis and prognosis.

## 6. Autologous Blood Infusion

### Question 10. Should intraoperative cell salvage (ICS) be routinely used to reduce allogeneic transfusion?

**Recommendation 16. Routine use of ICS is recommended in cardiac surgery (strong recommendation, high certainty)**.

ICS is the process of collecting the patient’s intraoperative blood loss, removing cell debris, free Hb, and activated procoagulant inflammatory products through procedure steps including filtration, centrifugal separation, and washing, and then reinfusing the blood to the patient [131]. Research has shown that using ICS during cardiac surgery can reduce the amount of allogeneic blood transfusions, lower the risk of transfusion-transmitted infections, and reduce hospitalization costs [132, 133, 134]. Meta-analysis suggests that ICS reduces the rate of allogeneic blood transfusion without increasing the requirement of fresh frozen plasma or platelets [135]. Therefore, it is recommended that ICS be used routinely.

### Question 11. Should autologous platelet-rich plasmapheresis (APP) be used before CPB to reduce allogenic transfusion?

**Recommendation 17. APP before CPB may be considered for patients with a high risk of post-CPB bleeding, stable hemodynamics, and normal platelet count and function (strong recommendation, moderate certainty)**.

CPB impairs the quality and quantity of platelets, especially in aortic surgery requiring deep hypothermic circulatory arrest. Meanwhile, separating and collecting the autologous platelets or platelet-rich plasma before CPB and reinfusing them after CPB is a way to preserve platelets. However, van der Wal *et al*. [136] demonstrated that this technique could neither reduce perioperative blood transfusion nor decrease blood loss in cardiac surgery. Triulzi *et al*. [137] found that although the postoperative blood loss was reduced, perioperative allogeneic blood transfusion was not lowered. In addition, collecting adequate platelets or platelet-rich plasma from approximately 20%–30% of the total blood volume might lead to hemodynamic instability, fluid overload, and severe hemodilution. Therefore, APP is not recommended in cardiac surgeries with low bleeding risk. As for surgeries with high bleeding risk, such as aortic surgery, multiple studies [138, 139, 140, 141] have shown that autologous platelet-rich plasma transfusion can improve patients’ coagulation function, reduce postoperative bleeding, and lower the incidence of pulmonary and renal complications.

### Question 12. Should acute normovolemic hemodilution (ANH) be used to reduce allogenic transfusion?

**Recommendation 18: ANH should be used in patients with stable conditions who anticipate prolonged CPB time or a high risk of bleeding (strong recommendation, moderate certainty)**.

ANH is a procedure in which a certain amount of autologous blood is collected from the patient after anesthesia induction and before systemic heparinization and then stored for later usage. At the same time, the crystalloid or colloid solutions are infused to replace the blood volume and achieve moderate hemodilution. The stored autologous blood will be transfused back to the patient after CPB or when the Hb level is below the transfusion threshold during CPB. ANH could preserve autologous blood from activation and damage by CPB. In 2017, a meta-analysis including 29 RCTs and 3439 patients showed that ANH can reduce the rate of perioperative allogeneic red blood cell transfusion and postoperative blood loss in adult cardiac surgery [142]. A meta-analysis in 2020 specifically focusing on ANH use in coronary artery bypass graft surgery also arrived at a similar conclusion [143]. The ANH volume is essential as research has shown that high-volume ANH (12–15 mL/kg or ≥800 mL) is more effective in reducing postoperative bleeding and perioperative blood product transfusion [144, 145]. Comparatively, large-volume ANH might increase the risk of hemodynamic instability during the ANH procedure, increase the degree of hemodilution, and lead to excessive fluid infusion. In addition, ANH should be performed before systemic heparization. The method is time-consuming and requires continuous, close hemodynamic monitoring, which is why ANH has not been widely implemented. There is no unified consensus on the optional candidate for ANH. However, it might be more suitable for patients with anticipated prolonged CPB time, high risk of bleeding, and stable hemodynamics. Thus, closely monitoring hemodynamics, Hb levels, and echocardiography is required during the implementation of ANH [146].

### Question 13. Should residual pump blood be transfused back into the patients?

**Recommendation 19. After CPB, the residual pump blood in the circuit should be transfused back into the patients (strong recommendation, high certainty)**.

Residual pump blood refers to the remnant blood in the circuit after weaning off CPB. A small-scale study showed that transfusing the residual pump blood back could increase Hb levels, platelet counts, and fibrinogen levels in the blood and improve the R-value , α angle, and maximum amplitude value of TEG [147]. The residual pump blood can be directly transfused or transfused after ultrafiltration or cell salvage processing. If the volume of residual pump blood is large, direct infusion will increase the patient’s fluid volume, while processing with ultrafiltration or ICS could reduce the volume. However, processing with ultrafiltration might increase plasma-free Hb levels, while processing with ICS might lead to the loss of plasma components and platelets. Some studies have compared the effects of three methods on allogeneic blood transfusion and coagulation function but have not yet reached consistent conclusions [148, 149, 150, 151, 152]. Although the optimal method for processing or not processing the residual pump blood has yet to be determined, it is widely recognized that residual pump blood is a valuable blood resource, and its transfusion after the end of the CPB is a critical component in the PBM program. These findings were supported by a study by Zhang *et al*. [9], which demonstrated a comprehensive PBM program, including residual blood pump infusion, could reduce the allogeneic transfusion rate.

## 7. Conclusion

Implementing a comprehensive PBM program in adult cardiac surgery under CPB could reduce blood loss, decrease allogeneic transfusion, and improve patient outcomes. These guidelines are focused on PBM measures related to CPB. During the development of these recommendations, the cost-effectiveness, acceptability, and feasibility of PBM measures in China were considered to facilitate a wide implementation of a comprehensive PBM program throughout China, aiming to promote perioperative PBM programs among hospitals at all levels. The comparisons between the 19 recommendations and those in other international guidelines are provided in **Supplementary Material D**. The guidelines issued by different regions and societies are generally similar in their core principles and recommendations. Furthermore, it is important to acknowledge that the evidence supporting certain aspects of these recommendations remains conflicting or insufficient. These knowledge gaps underscore the need for high-quality clinical research to strengthen the evidence base, including well-designed RCTs and large-scale observational studies.

## Abbreviations

CPB, cardiopulmonary bypass; PBM, patient blood management; AABB, Association for the Advancement of Blood and Biotherapies; PICO, population, intervention, comparison, and outcome; GRADE, Grading of Recommendations, Assessment, Development, and Evaluation; RCT, randomized controlled trial; RAP, retrograde autologous priming; ICU, intensive care unit; CUF, conventional ultrafiltration; MUF, modified ultrafiltration; AKI, acute kidney injury; ACT, activated clotting time; UFH, unfractionated heparin; ATⅢ, antithrombin Ⅲ; HMS, hemostatic management system; TEG, thromboelastogram; ROTEM, rotational thromboelastometry; POC, point-of-care; Hb, hemoglobin; DO_2_, oxygen delivery; SvO_2_, venous oxygen saturation; PCC, prothrombin complex concentrate; FFP, fresh frozen plasma; ICS, intraoperative cell salvage; APP, autologous platelet-rich plasmapheresis; ANH, acute normovolemic hemodilution.

## Supplementary Material

Supplementary material associated with this article can be found, in the online version, at https://doi.org/10.31083/RCM31384.
