# Supplementary material for: 2024 Guidelines on Patient Blood Management for Adult Cardiac Surgery Under Cardiopulmonary Bypass in China
Source: Rev Cardiovasc Med. 2025 Jun 16;26(6):31384. doi: 10.31083/RCM31384 (PMC12230837; doi:10.31083/RCM31384)
Supplement: Supplementary file 1 [file 2153-8174-26-6-31384-s1.docx]

**2024 Guidelines on Patient Blood Management for Adult Cardiac Surgery under Cardiopulmonary Bypass in China**

**Supplementary Materials**

[Supplementary Material A. Guideline Panel Members 2](#_Toc845519786)

[Supplementary Material B. PICO Questions and Literature Search Strategies 4](#_Toc751751103)

[Supplementary Material C. Examples for TEG/ROTEM Based Hemostatic Interventions and Transfusion Algorithms 37](#_Toc1034492820)

[Supplementary material D. Comparison between 2024 Guidelines on Patient Blood Management for Adult Cardiac Surgery under Cardiopulmonary Bypass in China and other International Guidelines 40](#_Toc693219628)

# Supplementary Material A. Guideline Panel Members

**(**Sorted alphabetically by the first letter of the Given Name）

**Chairpersons**: Bingyang Ji (Fuwai Hospital, Chinese Academy of Medical Sciences), Chengbin Zhou (Guangdong Provincial People's Hospital), Lei Du (West China Hospital, Sichuan University)

**Members:** Chen Chen (Guangdong Provincial People's Hospital), Chong'en Xu (Shandong Provincial Hospital), Guangcun Cheng (The First Affiliated Hospital of University of Science and Technology of China), Feilong Hei (Beijing Anzhen Hospital, Capital Medical University), Fuqing Jiang (Fuwai Hospital Shenzhen, Chinese Academy of Medical Sciences), Gang Liu (Fuwai Hospital, Chinese Academy of Medical Sciences), Guyan Wang (Beijing Tongren Hospital) , Guowei Tu (Zhongshan Hospital, Fudan University), Hongwen Ji (Fuwai Hospital, Chinese Academy of Medical Sciences), Hushan Ao (Fuwai Hospital, Chinese Academy of Medical Sciences), Jian Rong (The First Affiliated Hospital of Sun Yat-sen University), Jianhua Liu (Henan Provincial Chest Hospital), Jianxi Ye (Xiamen University Affiliated Cardiovascular Hospital), Jiaxin Ye (Nanjing Drum Tower Hospital), Jinping Liu (Fuwai Hospital, Chinese Academy of Medical Sciences), Jingyu Wang (The First Affiliated Hospital of Xi'an Jiaotong University), Jingjing Su (The First Affiliated Hospital of Harbin Medical University), Juan Xiao(Xinqiao Hospital, Army Medical University) Junbo Chuai (The Second Affiliated Hospital of Harbin Medical University), Jun Li (The First Affiliated Hospital of Zhengzhou University), Kai Liu (Qilu Hospital of Shandong University), Leiyi Yang (Fuwai Central China Cardiovascular Hospital), Li Zhou (West China Hospital, Sichuan University), Liping Shi (The First Affiliated Hospital of Medical School of Zhejiang University )，Liqiong Xiao (Nanjing First Hospital), Luyu Bian (Fuwai Hospital, Chinese Academy of Medical Sciences), Mingyue Liu (Zhongshan Hospital, Fudan University), Mingxia Zhao (Fuwai Hospital, Chinese Academy of Medical Sciences), Ping Li (Union Hospital, Tongji Medical College, Huazhong University of Science and Technology), Qiaoni Zhang(Fuwai Hospital, Chinese Academy of Medical Sciences), Renbin Tian (Affiliated Hospital of Zunyi Medical University), Ru Lin (Children's Hospital Zhejiang University School of Medicine), Sheng Wang (Beijing Anzhen Hospital, Capital Medical University), Shifu Wang (TEDA International Cardiovascular Hospital),Shujie Yan (Fuwai Hospital, Chinese Academy of Medical Sciences),Ting Wu (Tianjin Chest Hospital),Wei Wang (Shanghai Children's Medical Center, Shanghai Jiao Tong University School of Medicine),Xin Li (Zhongshan Hospital, Fudan University),Xuan Jiang (The First Affiliated Hospital of China Medical University),Yan Liu (Wuhan Asia Heart Hospital),Yaoyao Xiong (The Second Xiangya Hospital of Central South University),Yi Song (Yunnan Fuwai Cardiovascular Hospital),Yu Liu (Northern Theater Command General Hospital),Yuan Teng (Fuwai Hospital, Chinese Academy of Medical Sciences),Yuan Wang (Tongji Hospital, Tongji Medical College, Huazhong University of Science and Technology),Yongnian Liang (Jiangsu Provincial People's Hospital), Zhao Wang (Yan'an Hospital of Kunming),Zhen Guo (Shanghai Chest Hospital, Shanghai Jiao Tong University), Zhenxiao Jin (The First Affiliated Hospital of Air Force Medical University)

# Supplementary Material B. PICO Questions and Literature Search Strategies

**Question 1. Should the priming volume be reduced to decrease the perioperative allogeneic transfusions？**

***PICO 1a.***

(P)Adult cardiac surgery under cardiopulmonary bypass
(I) CPB circuit with the reduced priming volume; modified CPB circuit; mini-CPB; minimized CPB circuit

(C)Conventional priming volumes; conventional CPB circuit; conventional CPB
(O) RBC requirements, Bleeding; postoperative complications;

MEDLINE

#1 exp "Cardiac Surgical Procedures"[MeSH] OR exp "Coronary Artery Bypass"[MeSH] OR exp "Heart Valve Prosthesis Implantation"[MeSH] OR exp "Heart Transplantation"[MeSH] OR ("heart surgery"[TIAB] OR "cardiac surgery"[TIAB] OR "heart transplant*"[TIAB] OR "aortic surgery" [TIAB]OR "coronary artery bypass graft*" [TIAB]OR "valve replacement" [TIAB]OR "valve repair"[TIAB] OR "congenital heart surgery"[TIAB]）

#2 exp "Cardiopulmonary bypass"[MeSH] OR exp "Extracorporeal Circulation"[MeSH] OR ("cardiopulmonary bypass"[TIAB] OR "extracorporeal circulation"[TIAB])

#3 "priming volume"[TIAB] OR "Modified"[TIAB] OR "Mini"[TIAB] OR "Minimized"[TIAB] OR "miniaturized"[TIAB]

#4 #1 **AND** #2 **AND** #3

#5 #4 **AND** ("2000"[dp] : "3000"[dp]))

#6 exp "Adolescent"[MeSH] OR exp "Child"[MeSH] OR exp "Infant"[MeSH] OR ("Infant"[TI] OR"Pediatric"[TI] OR"Neonate*"[TI])

#7 "Review"[PT] OR"Editorial"[PT] OR "Comment"[PT] OR "Letter"[PT] OR "News"[PT] OR "Congress"[PT] OR "Published Erratum"[PT] OR"Case Reports"[PT]OR (book*[PT])

#5 NOT #6 NOT #7

Embase

#1 'heart surgery'/exp OR 'cardiovascular surgery'/exp OR 'aortic surgery'/exp OR ('heart surgery':ti,ab OR 'cardiac surgery':ti,ab OR 'heart transplant*':ti,ab OR 'aortic surgery':ti,ab OR 'coronary artery bypass graft*':ti,ab OR 'valve replacement':ti,ab OR 'valve repair':ti,ab OR 'congenital heart surgery':ti,ab)

#2 'cardiopulmonary bypass'/exp OR 'extracorporeal circulation'/exp OR ('cardiopulmonary bypass':ti,ab OR 'extracorporeal circulation':ti,ab)

#3 'priming volume':ti,ab OR 'modified':ti,ab OR 'mini':ti,ab OR 'minimized':ti,ab OR 'miniaturized':ti,ab

#4 #1 **AND** #2 **AND** #3

#5 #4 **AND** [2000-2024]/py

#6 'juvenile'/exp OR ('infant':ti OR 'pediatric':ti OR 'neonate*':ti)

#7 [Conference Abstract]/lim OR 'editorial'/it OR 'letter'/it OR 'note'/it OR 'chapter'/it OR 'conference abstract'/it OR 'conference review'/it OR 'editorial'/it OR 'erratum'/it OR [preprint]/lim OR 'case study'/exp OR 'case report'/exp OR (((case*) NEAR/3 (report* OR stud*)) OR comment):ti

#5 NOT #6 NOT #7

***PICO 1b.***

(P)Adult cardiac surgery under cardiopulmonary bypass
(I) retrograde autologous priming

(C) no retrograde autologous priming

(O) RBC requirements, Bleeding; postoperative complications;

MEDLINE

#1 exp "Cardiac Surgical Procedures"[MeSH] OR exp "Coronary Artery Bypass"[MeSH] OR exp "Heart Valve Prosthesis Implantation"[MeSH] OR exp "Heart Transplantation"[MeSH] OR ("heart surgery"[TIAB] OR "cardiac surgery"[TIAB] OR "heart transplant*"[TIAB] OR "aortic surgery" [TIAB]OR "coronary artery bypass graft*" [TIAB]OR "valve replacement" [TIAB]OR "valve repair"[TIAB] OR "congenital heart surgery"[TIAB]）

#2 exp "Cardiopulmonary bypass"[MeSH] OR exp "Extracorporeal Circulation"[MeSH] OR ("cardiopulmonary bypass"[TIAB] OR "extracorporeal circulation"[TIAB])

#3 "retrograde autologous priming"[TIAB] OR "autologous priming"[TIAB]

#4 #1 **AND** #2 **AND** #3

#5 #4 **AND** ("2000"[dp] : "3000"[dp]))

#6 exp "Adolescent"[MeSH] OR exp "Child"[MeSH] OR exp "Infant"[MeSH] OR ("Infant"[TI] OR"Pediatric"[TI] OR"Neonate*"[TI])

#7 "Review"[PT] OR"Editorial"[PT] OR "Comment"[PT] OR "Letter"[PT] OR "News"[PT] OR "Congress"[PT] OR "Published Erratum"[PT] OR"Case Reports"[PT]OR (book*[PT])

#5 NOT #6 NOT #7

Embase

#1 'heart surgery'/exp OR 'cardiovascular surgery'/exp OR 'aortic surgery'/exp OR ('heart surgery':ti,ab OR 'cardiac surgery':ti,ab OR 'heart transplant*':ti,ab OR 'aortic surgery':ti,ab OR 'coronary artery bypass graft*':ti,ab OR 'valve replacement':ti,ab OR 'valve repair':ti,ab OR 'congenital heart surgery':ti,ab)

#2 'cardiopulmonary bypass'/exp OR 'extracorporeal circulation'/exp OR ('cardiopulmonary bypass':ti,ab OR 'extracorporeal circulation':ti,ab)

#3 'retrograde autologous priming':ti,ab OR 'autologous priming':ti,ab

#4 #1 **AND** #2 **AND** #3

#5 #4 **AND** [2000-2024]/py

#6 'juvenile'/exp OR ('infant':ti OR 'pediatric':ti OR 'neonate*':ti)

#7 [Conference Abstract]/lim OR 'editorial'/it OR 'letter'/it OR 'note'/it OR 'chapter'/it OR 'conference abstract'/it OR 'conference review'/it OR 'editorial'/it OR 'erratum'/it OR [preprint]/lim OR 'case study'/exp OR 'case report'/exp OR (((case*) NEAR/3 (report* OR stud*)) OR comment):ti

#5 NOT #6 NOT #7

**Question 2. Which solutions should be selected as the optimal priming solution？**

***PICO 2a.***

1. Adult cardiac surgery under cardiopulmonary bypass
   (I) Balanced crystalloid solutions
2. 0.9% saline
3. RBC requirements, Bleeding; postoperative complications;

MEDLINE

#1 exp "Cardiac Surgical Procedures"[MeSH] OR exp "Coronary Artery Bypass"[MeSH] OR exp "Heart Valve Prosthesis Implantation"[MeSH] OR exp "Heart Transplantation"[MeSH] OR ("heart surgery"[TIAB] OR "cardiac surgery"[TIAB] OR "heart transplant*"[TIAB] OR "aortic surgery" [TIAB]OR "coronary artery bypass graft*" [TIAB]OR "valve replacement" [TIAB]OR "valve repair"[TIAB] OR "congenital heart surgery"[TIAB]）

#2 exp "Cardiopulmonary bypass"[MeSH] OR exp "Extracorporeal Circulation"[MeSH] OR ("cardiopulmonary bypass"[TIAB] OR "extracorporeal circulation"[TIAB])

#3 "balanced crystalloid*"[TIAB] OR "cystalloid*"[TIAB]

#4 #1 AND #2 AND #3

#5 #4 AND ("2000"[dp] : "3000"[dp]))

#6 exp "Adolescent"[MeSH] OR exp "Child"[MeSH] OR exp "Infant"[MeSH] OR ("Infant"[TI] OR"Pediatric"[TI] OR"Neonate*"[TI])

#7 "Review"[PT] OR"Editorial"[PT] OR "Comment"[PT] OR "Letter"[PT] OR "News"[PT] OR "Congress"[PT] OR "Published Erratum"[PT] OR"Case Reports"[PT]OR (book*[PT])

#5 NOT #6 NOT #7

Embase

#1 'heart surgery'/exp OR 'cardiovascular surgery'/exp OR 'aortic surgery'/exp OR ('heart surgery':ti,ab OR 'cardiac surgery':ti,ab OR 'heart transplant*':ti,ab OR 'aortic surgery':ti,ab OR 'coronary artery bypass graft*':ti,ab OR 'valve replacement':ti,ab OR 'valve repair':ti,ab OR 'congenital heart surgery':ti,ab)

#2 'cardiopulmonary bypass'/exp OR 'extracorporeal circulation'/exp OR ('cardiopulmonary bypass':ti,ab OR 'extracorporeal circulation':ti,ab)

#3 'balanced crystalloid*':ti,ab OR 'crystalloid*':ti,ab

#4 #1 AND #2 AND #3

#5 #4 AND [2000-2024]/py

#6 'juvenile'/exp OR ('infant':ti OR 'pediatric':ti OR 'neonate*':ti)

#7 [Conference Abstract]/lim OR 'editorial'/it OR 'letter'/it OR 'note'/it OR 'chapter'/it OR 'conference abstract'/it OR 'conference review'/it OR 'editorial'/it OR 'erratum'/it OR [preprint]/lim OR 'case study'/exp OR 'case report'/exp OR (((case*) NEAR/3 (report* OR stud*)) OR comment):ti

#5 NOT #6 NOT #7

***PICO 2b.***

1. Adult cardiac surgery under cardiopulmonary bypass
   (I) albumin solution
2. crystalloid OR Hydroxyethyl starch OR Gelatin solutions
3. RBC requirements, Bleeding; postoperative complications;

MEDLINE

#1 exp "Cardiac Surgical Procedures"[MeSH] OR exp "Coronary Artery Bypass"[MeSH] OR exp "Heart Valve Prosthesis Implantation"[MeSH] OR exp "Heart Transplantation"[MeSH] OR ("heart surgery"[TIAB] OR "cardiac surgery"[TIAB] OR "heart transplant*"[TIAB] OR "aortic surgery" [TIAB]OR "coronary artery bypass graft*" [TIAB]OR "valve replacement" [TIAB]OR "valve repair"[TIAB] OR "congenital heart surgery"[TIAB] OR "hypoalbuminemia"[TIAB] ）

#2 exp "Cardiopulmonary bypass"[MeSH] OR exp "Extracorporeal Circulation"[MeSH] OR ("cardiopulmonary bypass"[TIAB] OR "extracorporeal circulation"[TIAB])

#3 "human albumin solution*"[TIAB] OR "albumin solution*"[TIAB]

#4 #1 AND #2 AND #3

#5 #4 AND ("2000"[dp] : "3000"[dp]))

#6 exp "Adolescent"[MeSH] OR exp "Child"[MeSH] OR exp "Infant"[MeSH] OR ("Infant"[TI] OR"Pediatric"[TI] OR"Neonate*"[TI])

#7 "Review"[PT] OR"Editorial"[PT] OR "Comment"[PT] OR "Letter"[PT] OR "News"[PT] OR "Congress"[PT] OR "Published Erratum"[PT] OR"Case Reports"[PT]OR (book*[PT])

#5 NOT #6 NOT #7

Embase

#1 'heart surgery'/exp OR 'cardiovascular surgery'/exp OR 'aortic surgery'/exp OR ('heart surgery':ti,ab OR 'cardiac surgery':ti,ab OR 'heart transplant*':ti,ab OR 'aortic surgery':ti,ab OR 'coronary artery bypass graft*':ti,ab OR 'valve replacement':ti,ab OR 'valve repair':ti,ab OR 'congenital heart surgery':ti,ab OR 'hypoalbuminemia':ti,ab )

#2 'cardiopulmonary bypass'/exp OR 'extracorporeal circulation'/exp OR ('cardiopulmonary bypass':ti,ab OR 'extracorporeal circulation':ti,ab)

#3 'human albumin solution*':ti,ab OR 'albumin solution*':ti,ab

#4 #1 AND #2 AND #3

#5 #4 AND [2000-2024]/py

#6 'juvenile'/exp OR ('infant':ti OR 'pediatric':ti OR 'neonate*':ti)

#7 [Conference Abstract]/lim OR 'editorial'/it OR 'letter'/it OR 'note'/it OR 'chapter'/it OR 'conference abstract'/it OR 'conference review'/it OR 'editorial'/it OR 'erratum'/it OR [preprint]/lim OR 'case study'/exp OR 'case report'/exp OR (((case*) NEAR/3 (report* OR stud*)) OR comment):ti

#5 NOT #6 NOT #7

**Question 3. Should ultrafiltration be used during CPB to reduce allogeneic transfusion？**

***PICO 3.***

(P)Adult cardiac surgery under cardiopulmonary bypass
(I) ultrafiltration

(C) no ultrafiltration

(O)RBC requirements, Bleeding; acute kidney injury; postoperative complications;

MEDLINE

#1 exp "Cardiac Surgical Procedures"[MeSH] OR exp "Coronary Artery Bypass"[MeSH] OR exp "Heart Valve Prosthesis Implantation"[MeSH] OR exp "Heart Transplantation"[MeSH] OR ("heart surgery"[TIAB] OR "cardiac surgery"[TIAB] OR "heart transplant*"[TIAB] OR "aortic surgery" [TIAB]OR "coronary artery bypass graft*" [TIAB]OR "valve replacement" [TIAB]OR "valve repair"[TIAB] OR "congenital heart surgery"[TIAB]）

#2 exp "Cardiopulmonary bypass"[MeSH] OR exp "Extracorporeal Circulation"[MeSH] OR ("cardiopulmonary bypass"[TIAB] OR "extracorporeal circulation"[TIAB])

#3 "ultrafiltration"[TIAB]

#4 #1 AND #2 AND #3

#5 #4 AND ("2000"[dp] : "3000"[dp]))

#6 exp "Adolescent"[MeSH] OR exp "Child"[MeSH] OR exp "Infant"[MeSH] OR ("Infant"[TI] OR"Pediatric"[TI] OR"Neonate*"[TI])

#7 "Review"[PT] OR"Editorial"[PT] OR "Comment"[PT] OR "Letter"[PT] OR "News"[PT] OR "Congress"[PT] OR "Published Erratum"[PT] OR"Case Reports"[PT]OR (book*[PT])

#5 NOT #6 NOT #7

Embase

#1 'heart surgery'/exp OR 'cardiovascular surgery'/exp OR 'aortic surgery'/exp OR ('heart surgery':ti,ab OR 'cardiac surgery':ti,ab OR 'heart transplant*':ti,ab OR 'aortic surgery':ti,ab OR 'coronary artery bypass graft*':ti,ab OR 'valve replacement':ti,ab OR 'valve repair':ti,ab OR 'congenital heart surgery':ti,ab)

#2 'cardiopulmonary bypass'/exp OR 'extracorporeal circulation'/exp OR ('cardiopulmonary bypass':ti,ab OR 'extracorporeal circulation':ti,ab)

#3 'ultrafiltration':ti,ab

#4 #1 AND #2 AND #3

#5 #4 AND [2000-2024]/py

#6 'juvenile'/exp OR ('infant':ti OR 'pediatric':ti OR 'neonate*':ti)

#7 [Conference Abstract]/lim OR 'editorial'/it OR 'letter'/it OR 'note'/it OR 'chapter'/it OR 'conference abstract'/it OR 'conference review'/it OR 'editorial'/it OR 'erratum'/it OR [preprint]/lim OR 'case study'/exp OR 'case report'/exp OR (((case*) NEAR/3 (report* OR stud*)) OR comment):ti

#5 NOT #6 NOT #7

**Question 4: How should heparization and protamine neutralization be managed appropriately?**

***PICO 4a.***

1. Adult cardiac surgery under cardiopulmonary bypass; heparin resistance
   (I) fresh frozen plasma

(C) no intervention; AT concentrates

(O) RBC requirements, Bleeding; manage heparin resistence; postoperative complications;

MEDLINE

#1 exp "Cardiac Surgical Procedures"[MeSH] OR exp "Coronary Artery Bypass"[MeSH] OR exp "Heart Valve Prosthesis Implantation"[MeSH] OR exp "Heart Transplantation"[MeSH] OR ("heart surgery"[TIAB] OR "cardiac surgery"[TIAB] OR "heart transplant*"[TIAB] OR "aortic surgery" [TIAB]OR "coronary artery bypass graft*" [TIAB]OR "valve replacement" [TIAB]OR "valve repair"[TIAB] OR "congenital heart surgery"[TIAB]）

#2 exp "Cardiopulmonary bypass"[MeSH] OR exp "Extracorporeal Circulation"[MeSH] OR ("cardiopulmonary bypass"[TIAB] OR "extracorporeal circulation"[TIAB])

#3 "heparin resistence"[TIAB]

#4 #1 AND #2 AND #3

#5 #4 AND ("2000"[dp] : "3000"[dp]))

#6 exp "Adolescent"[MeSH] OR exp "Child"[MeSH] OR exp "Infant"[MeSH] OR ("Infant"[TI] OR"Pediatric"[TI] OR"Neonate*"[TI])

#7 "Review"[PT] OR"Editorial"[PT] OR "Comment"[PT] OR "Letter"[PT] OR "News"[PT] OR "Congress"[PT] OR "Published Erratum"[PT] OR"Case Reports"[PT]OR (book*[PT])

#5 NOT #6 NOT #7

Embase

#1 'heart surgery'/exp OR 'cardiovascular surgery'/exp OR 'aortic surgery'/exp OR ('heart surgery':ti,ab OR 'cardiac surgery':ti,ab OR 'heart transplant*':ti,ab OR 'aortic surgery':ti,ab OR 'coronary artery bypass graft*':ti,ab OR 'valve replacement':ti,ab OR 'valve repair':ti,ab OR 'congenital heart surgery':ti,ab)

#2 'cardiopulmonary bypass'/exp OR 'extracorporeal circulation'/exp OR ('cardiopulmonary bypass':ti,ab OR 'extracorporeal circulation':ti,ab)

#3 'heparin resistence':ti,ab

#4 #1 AND #2 AND #3

#5 #4 AND [2000-2024]/py

#6 'juvenile'/exp OR ('infant':ti OR 'pediatric':ti OR 'neonate*':ti)

#7 [Conference Abstract]/lim OR 'editorial'/it OR 'letter'/it OR 'note'/it OR 'chapter'/it OR 'conference abstract'/it OR 'conference review'/it OR 'editorial'/it OR 'erratum'/it OR [preprint]/lim OR 'case study'/exp OR 'case report'/exp OR (((case*) NEAR/3 (report* OR stud*)) OR comment):ti

#5 NOT #6 NOT #7

***PICO 4b.***

(P)Adult cardiac surgery under cardiopulmonary bypass;
(I) heparin dosing ; protamine dosing

1. conventional

(O) RBC requirements, Bleeding; postoperative complications;

MEDLINE

#1 exp "Cardiac Surgical Procedures"[MeSH] OR exp "Coronary Artery Bypass"[MeSH] OR exp "Heart Valve Prosthesis Implantation"[MeSH] OR exp "Heart Transplantation"[MeSH] OR ("heart surgery"[TIAB] OR "cardiac surgery"[TIAB] OR "heart transplant*"[TIAB] OR "aortic surgery" [TIAB]OR "coronary artery bypass graft*" [TIAB]OR "valve replacement" [TIAB]OR "valve repair"[TIAB] OR "congenital heart surgery"[TIAB]）

#2 exp "Cardiopulmonary bypass"[MeSH] OR exp "Extracorporeal Circulation"[MeSH] OR ("cardiopulmonary bypass"[TIAB] OR "extracorporeal circulation"[TIAB])

#3 "heparin dos*"[TIAB] OR "protamine dos*"[TIAB] OR "protamine titation"[TIAB] OR "protamine overdos*"[TIAB] OR "heparin rebound"[TIAB]

#4 #1 AND #2 AND #3

#5 #4 AND ("2000"[dp] : "3000"[dp]))

#6 exp "Adolescent"[MeSH] OR exp "Child"[MeSH] OR exp "Infant"[MeSH] OR ("Infant"[TI] OR"Pediatric"[TI] OR"Neonate*"[TI])

#7 "Review"[PT] OR"Editorial"[PT] OR "Comment"[PT] OR "Letter"[PT] OR "News"[PT] OR "Congress"[PT] OR "Published Erratum"[PT] OR"Case Reports"[PT]OR (book*[PT])

#5 NOT #6 NOT #7

Embase

#1 'heart surgery'/exp OR 'cardiovascular surgery'/exp OR 'aortic surgery'/exp OR ('heart surgery':ti,ab OR 'cardiac surgery':ti,ab OR 'heart transplant*':ti,ab OR 'aortic surgery':ti,ab OR 'coronary artery bypass graft*':ti,ab OR 'valve replacement':ti,ab OR 'valve repair':ti,ab OR 'congenital heart surgery':ti,ab)

#2 'cardiopulmonary bypass'/exp OR 'extracorporeal circulation'/exp OR ('cardiopulmonary bypass':ti,ab OR 'extracorporeal circulation':ti,ab)

#3 'heparin dos*':ti,ab OR 'protamine dos*':ti,ab OR 'protamine titation':ti,ab OR 'protamine overdos*':ti,ab OR 'heparin rebound':ti,ab

#4 #1 AND #2 AND #3

#5 #4 AND [2000-2024]/py

#6 'juvenile'/exp OR ('infant':ti OR 'pediatric':ti OR 'neonate*':ti)

#7 [Conference Abstract]/lim OR 'editorial'/it OR 'letter'/it OR 'note'/it OR 'chapter'/it OR 'conference abstract'/it OR 'conference review'/it OR 'editorial'/it OR 'erratum'/it OR [preprint]/lim OR 'case study'/exp OR 'case report'/exp OR (((case*) NEAR/3 (report* OR stud*)) OR comment):ti

#5 NOT #6 NOT #7

**Question 5: How should anticoagulation be appropriately monitored during CPB?**

***PICO 5.***

(P)Adult cardiac surgery under cardiopulmonary bypass;
(I) ACT above 480

(C) other threshold

(O) RBC requirements, Bleeding; thrombosis; postoperative complications;

MEDLINE

#1 exp "Cardiac Surgical Procedures"[MeSH] OR exp "Coronary Artery Bypass"[MeSH] OR exp "Heart Valve Prosthesis Implantation"[MeSH] OR exp "Heart Transplantation"[MeSH] OR ("heart surgery"[TIAB] OR "cardiac surgery"[TIAB] OR "heart transplant*"[TIAB] OR "aortic surgery" [TIAB]OR "coronary artery bypass graft*" [TIAB]OR "valve replacement" [TIAB]OR "valve repair"[TIAB] OR "congenital heart surgery"[TIAB]）

#2 exp "Cardiopulmonary bypass"[MeSH] OR exp "Extracorporeal Circulation"[MeSH] OR ("cardiopulmonary bypass"[TIAB] OR "extracorporeal circulation"[TIAB])

#3 "heparin monitor*"[TIAB] OR "ACT"[TIAB] OR "Activated clot* time"[TIAB] #4 #1 AND #2 AND #3

#5 #4 AND ("2000"[dp] : "3000"[dp]))

#6 exp "Adolescent"[MeSH] OR exp "Child"[MeSH] OR exp "Infant"[MeSH] OR ("Infant"[TI] OR"Pediatric"[TI] OR"Neonate*"[TI])

#7 "Review"[PT] OR"Editorial"[PT] OR "Comment"[PT] OR "Letter"[PT] OR "News"[PT] OR "Congress"[PT] OR "Published Erratum"[PT] OR"Case Reports"[PT]OR (book*[PT])

#5 NOT #6 NOT #7

Embase

#1 'heart surgery'/exp OR 'cardiovascular surgery'/exp OR 'aortic surgery'/exp OR ('heart surgery':ti,ab OR 'cardiac surgery':ti,ab OR 'heart transplant*':ti,ab OR 'aortic surgery':ti,ab OR 'coronary artery bypass graft*':ti,ab OR 'valve replacement':ti,ab OR 'valve repair':ti,ab OR 'congenital heart surgery':ti,ab)

#2 'cardiopulmonary bypass'/exp OR 'extracorporeal circulation'/exp OR ('cardiopulmonary bypass':ti,ab OR 'extracorporeal circulation':ti,ab)

#3 'heparin monitor*':ti,ab OR 'ACT':ti,ab OR 'Activated clot* time':ti,ab

#4 #1 AND #2 AND #3

#5 #4 AND [2000-2024]/py

#6 'juvenile'/exp OR ('infant':ti OR 'pediatric':ti OR 'neonate*':ti)

#7 [Conference Abstract]/lim OR 'editorial'/it OR 'letter'/it OR 'note'/it OR 'chapter'/it OR 'conference abstract'/it OR 'conference review'/it OR 'editorial'/it OR 'erratum'/it OR [preprint]/lim OR 'case study'/exp OR 'case report'/exp OR (((case*) NEAR/3 (report* OR stud*)) OR comment):ti

#5 NOT #6 NOT #7

**Question 6: Should viscoelastic testing be performed to guide hemostasis management and reduce bleeding?**

***PICO 6.***

(P)Adult cardiac surgery under cardiopulmonary bypass;
(I) viscoelastic testing; TEG; ROTEM

(C）not use

(O) RBC requirements, Bleeding; thrombosis; postoperative complications;

MEDLINE

#1 exp "Cardiac Surgical Procedures"[MeSH] OR exp "Coronary Artery Bypass"[MeSH] OR exp "Heart Valve Prosthesis Implantation"[MeSH] OR exp "Heart Transplantation"[MeSH] OR ("heart surgery"[TIAB] OR "cardiac surgery"[TIAB] OR "heart transplant*"[TIAB] OR "aortic surgery" [TIAB]OR "coronary artery bypass graft*" [TIAB]OR "valve replacement" [TIAB]OR "valve repair"[TIAB] OR "congenital heart surgery"[TIAB]）

#2 exp "Cardiopulmonary bypass"[MeSH] OR exp "Extracorporeal Circulation"[MeSH] OR ("cardiopulmonary bypass"[TIAB] OR "extracorporeal circulation"[TIAB])

#3 "viscoelastic"[TIAB] OR "TEG"[TIAB] OR "ROTEM"[TIAB] OR "thromboelastography"[TIAB] OR "thromboelastometry"[TIAB] OR"point of care"[TIAB]

#4 #1 AND #2 AND #3

#5 #4 AND ("2000"[dp] : "3000"[dp]))

#6 exp "Adolescent"[MeSH] OR exp "Child"[MeSH] OR exp "Infant"[MeSH] OR ("Infant"[TI] OR"Pediatric"[TI] OR"Neonate*"[TI])

#7 "Review"[PT] OR"Editorial"[PT] OR "Comment"[PT] OR "Letter"[PT] OR "News"[PT] OR "Congress"[PT] OR "Published Erratum"[PT] OR"Case Reports"[PT]OR (book*[PT])

#5 NOT #6 NOT #7

Embase

#1 'heart surgery'/exp OR 'cardiovascular surgery'/exp OR 'aortic surgery'/exp OR ('heart surgery':ti,ab OR 'cardiac surgery':ti,ab OR 'heart transplant*':ti,ab OR 'aortic surgery':ti,ab OR 'coronary artery bypass graft*':ti,ab OR 'valve replacement':ti,ab OR 'valve repair':ti,ab OR 'congenital heart surgery':ti,ab)

#2 'cardiopulmonary bypass'/exp OR 'extracorporeal circulation'/exp OR ('cardiopulmonary bypass':ti,ab OR 'extracorporeal circulation':ti,ab)

#3 'viscoelastic':ti,ab OR 'TEG':ti,ab OR 'ROTEM':ti,ab OR 'thromboelastography':ti,ab OR 'thromboelastometry':ti,ab OR 'point of care':ti,ab

#4 #1 AND #2 AND #3

#5 #4 AND [2000-2024]/py

#6 'juvenile'/exp OR ('infant':ti OR 'pediatric':ti OR 'neonate*':ti)

#7 [Conference Abstract]/lim OR 'editorial'/it OR 'letter'/it OR 'note'/it OR 'chapter'/it OR 'conference abstract'/it OR 'conference review'/it OR 'editorial'/it OR 'erratum'/it OR [preprint]/lim OR 'case study'/exp OR 'case report'/exp OR (((case*) NEAR/3 (report* OR stud*)) OR comment):ti

#5 NOT #6 NOT #7

**Question 7: Should antifibrinolytic therapy be used to reduce bleeding and transfusion?**

***PICO 7.***

(P)Adult cardiac surgery under cardiopulmonary bypass;
(I) antifibrinolytic therapy; Tranexamic acid; Epsilon aminocaproic acid

(C）not use

(O) RBC requirements, Bleeding; seizure; postoperative complications;

MEDLINE

#1 exp "Cardiac Surgical Procedures"[MeSH] OR exp "Coronary Artery Bypass"[MeSH] OR exp "Heart Valve Prosthesis Implantation"[MeSH] OR exp "Heart Transplantation"[MeSH] OR ("heart surgery"[TIAB] OR "cardiac surgery"[TIAB] OR "heart transplant*"[TIAB] OR "aortic surgery" [TIAB]OR "coronary artery bypass graft*" [TIAB]OR "valve replacement" [TIAB]OR "valve repair"[TIAB] OR "congenital heart surgery"[TIAB]）

#2 exp "Cardiopulmonary bypass"[MeSH] OR exp "Extracorporeal Circulation"[MeSH] OR ("cardiopulmonary bypass"[TIAB] OR "extracorporeal circulation"[TIAB])

#3 "antifibrinolytic"[TIAB] OR "tranexamic acid"[TIAB] OR "aminocaproic acid"[TIAB] OR "epsilon aminocaproic acid"[TIAB]

#4 #1 AND #2 AND #3

#5 #4 AND ("2000"[dp] : "3000"[dp]))

#6 exp "Adolescent"[MeSH] OR exp "Child"[MeSH] OR exp "Infant"[MeSH] OR ("Infant"[TI] OR"Pediatric"[TI] OR"Neonate*"[TI])

#7 "Review"[PT] OR"Editorial"[PT] OR "Comment"[PT] OR "Letter"[PT] OR "News"[PT] OR "Congress"[PT] OR "Published Erratum"[PT] OR"Case Reports"[PT]OR (book*[PT])

#5 NOT #6 NOT #7

Embase

#1 'heart surgery'/exp OR 'cardiovascular surgery'/exp OR 'aortic surgery'/exp OR ('heart surgery':ti,ab OR 'cardiac surgery':ti,ab OR 'heart transplant*':ti,ab OR 'aortic surgery':ti,ab OR 'coronary artery bypass graft*':ti,ab OR 'valve replacement':ti,ab OR 'valve repair':ti,ab OR 'congenital heart surgery':ti,ab)

#2 'cardiopulmonary bypass'/exp OR 'extracorporeal circulation'/exp OR ('cardiopulmonary bypass':ti,ab OR 'extracorporeal circulation':ti,ab)

#3 'antifibrinolytic':ti,ab OR 'tranexamic acid':ti,ab OR 'aminocaproic acid':ti,ab OR 'epsilon aminocaproic acid':ti,ab

#4 #1 AND #2 AND #3

#5 #4 AND [2000-2024]/py

#6 'juvenile'/exp OR ('infant':ti OR 'pediatric':ti OR 'neonate*':ti)

#7 [Conference Abstract]/lim OR 'editorial'/it OR 'letter'/it OR 'note'/it OR 'chapter'/it OR 'conference abstract'/it OR 'conference review'/it OR 'editorial'/it OR 'erratum'/it OR [preprint]/lim OR 'case study'/exp OR 'case report'/exp OR (((case*) NEAR/3 (report* OR stud*)) OR comment):ti

#5 NOT #6 NOT #7

**Question 8: Should a restrictive transfusion strategy be applied in cardiac surgery?**

***PICO 8.***

(P)Adult cardiac surgery under cardiopulmonary bypass;
(I) restrictive transfusion

(C）liberal transfusion strategy

(O) transfusion; mortality; postoperative complications;

MEDLINE

#1 exp "Cardiac Surgical Procedures"[MeSH] OR exp "Coronary Artery Bypass"[MeSH] OR exp "Heart Valve Prosthesis Implantation"[MeSH] OR exp "Heart Transplantation"[MeSH] OR ("heart surgery"[TIAB] OR "cardiac surgery"[TIAB] OR "heart transplant*"[TIAB] OR "aortic surgery" [TIAB]OR "coronary artery bypass graft*" [TIAB]OR "valve replacement" [TIAB]OR "valve repair"[TIAB] OR "congenital heart surgery"[TIAB]）

#2 exp "Cardiopulmonary bypass"[MeSH] OR exp "Extracorporeal Circulation"[MeSH] OR ("cardiopulmonary bypass"[TIAB] OR "extracorporeal circulation"[TIAB])

#3 exp "blood transfusion"[MeSH] OR "erythrocyte transfusion"[MeSH] OR "red blood cell transfusion*"[TIAB] OR "RBC transfusion"[TIAB] OR "pRBC transfusion"[TIAB]

#4 #1 AND #2 AND #3

#5 #4 AND ("2000"[dp] : "3000"[dp]))

#6 exp "Adolescent"[MeSH] OR exp "Child"[MeSH] OR exp "Infant"[MeSH] OR ("Infant"[TI] OR"Pediatric"[TI] OR"Neonate*"[TI])

#7 "Review"[PT] OR"Editorial"[PT] OR "Comment"[PT] OR "Letter"[PT] OR "News"[PT] OR "Congress"[PT] OR "Published Erratum"[PT] OR"Case Reports"[PT]OR (book*[PT])

#5 NOT #6 NOT #7

Embase

#1 'heart surgery'/exp OR 'cardiovascular surgery'/exp OR 'aortic surgery'/exp OR ('heart surgery':ti,ab OR 'cardiac surgery':ti,ab OR 'heart transplant*':ti,ab OR 'aortic surgery':ti,ab OR 'coronary artery bypass graft*':ti,ab OR 'valve replacement':ti,ab OR 'valve repair':ti,ab OR 'congenital heart surgery':ti,ab)

#2 'cardiopulmonary bypass'/exp OR 'extracorporeal circulation'/exp OR ('cardiopulmonary bypass':ti,ab OR 'extracorporeal circulation':ti,ab)

#3 'blood transfusion'/exp OR 'erythrocyte transfusion'/exp OR 'red blood cell transfusion':ti,ab OR 'RBC transfusion':ti,ab OR 'pRBC transfusion':ti,ab

#4 #1 AND #2 AND #3

#5 #4 AND [2000-2024]/py

#6 'juvenile'/exp OR ('infant':ti OR 'pediatric':ti OR 'neonate*':ti)

#7 [Conference Abstract]/lim OR 'editorial'/it OR 'letter'/it OR 'note'/it OR 'chapter'/it OR 'conference abstract'/it OR 'conference review'/it OR 'editorial'/it OR 'erratum'/it OR [preprint]/lim OR 'case study'/exp OR 'case report'/exp OR (((case*) NEAR/3 (report* OR stud*)) OR comment):ti

#5 NOT #6 NOT #7

**Question 9: How should other blood products be used appropriately?**

***PICO 9a.***

(P)Adult cardiac surgery with severe bleeding after cardiopulmonary bypass;
(I) prothrombin complex concentrate

(C）fresh frozen plasma; no use

(O) transfusion; bleeding; postoperative complications;

MEDLINE

#1 exp "Cardiac Surgical Procedures"[MeSH] OR exp "Coronary Artery Bypass"[MeSH] OR exp "Heart Valve Prosthesis Implantation"[MeSH] OR exp "Heart Transplantation"[MeSH] OR ("heart surgery"[TIAB] OR "cardiac surgery"[TIAB] OR "heart transplant*"[TIAB] OR "aortic surgery" [TIAB]OR "coronary artery bypass graft*" [TIAB]OR "valve replacement" [TIAB]OR "valve repair"[TIAB] OR "congenital heart surgery"[TIAB]）

#2 exp "Cardiopulmonary bypass"[MeSH] OR exp "Extracorporeal Circulation"[MeSH] OR ("cardiopulmonary bypass"[TIAB] OR "extracorporeal circulation"[TIAB])

#3 exp "hemorrhage"[MeSH] OR exp "blood loss, surgical"[MeSH] OR exp "blood coagulation disorders"[MeSH] OR exp "hemostasis"[MeSH] OR "hemostasis"[TIAB] OR "blood loss"[TIAB] OR "hemorrhage"[TIAB] OR "coagulopathy"[TIAB]

#4 "prothrombin"[TIAB] OR "PCC"[TIAB] OR "prothrombin **complex concentrate**"[TIAB] OR "plasma transfusion"[TIAB] OR "FFP"[TIAB]

#5 #1 AND #2 AND #3 AND #4

#6 #5 AND ("2000"[dp] : "3000"[dp]))

#7 exp "Adolescent"[MeSH] OR exp "Child"[MeSH] OR exp "Infant"[MeSH] OR ("Infant"[TI] OR"Pediatric"[TI] OR"Neonate*"[TI])

#8 "Review"[PT] OR"Editorial"[PT] OR "Comment"[PT] OR "Letter"[PT] OR "News"[PT] OR "Congress"[PT] OR "Published Erratum"[PT] OR"Case Reports"[PT]OR (book*[PT])

#6 NOT #7 NOT #8

Embase

#1 'heart surgery'/exp OR 'cardiovascular surgery'/exp OR 'aortic surgery'/exp OR ('heart surgery':ti,ab OR 'cardiac surgery':ti,ab OR 'heart transplant*':ti,ab OR 'aortic surgery':ti,ab OR 'coronary artery bypass graft*':ti,ab OR 'valve replacement':ti,ab OR 'valve repair':ti,ab OR 'congenital heart surgery':ti,ab)

#2 'cardiopulmonary bypass'/exp OR 'extracorporeal circulation'/exp OR ('cardiopulmonary bypass':ti,ab OR 'extracorporeal circulation':ti,ab)

#3 'bleeding'/exp OR 'hemostasis'/exp OR 'blood loss':ti,ab OR 'hemorrhage':ti,ab OR 'bleeding':ti,ab OR 'coagulopathy':ti,ab

#4 'prothrombin complex'/exp OR 'plasma transfusion'/exp OR 'prothrombin':ti,ab OR 'prothrombin':ti,ab OR 'PCC':ti,ab OR 'prothrombin complex concentrate':ti,ab OR 'plasma transfusion':ti,ab OR 'FFP':ti,ab

#5 #1 AND #2 AND #3 AND #4

#6 #5 AND [2000-2024]/py

#7 'juvenile'/exp OR ('infant':ti OR 'pediatric':ti OR 'neonate*':ti)

#8 [Conference Abstract]/lim OR 'editorial'/it OR 'letter'/it OR 'note'/it OR 'chapter'/it OR 'conference abstract'/it OR 'conference review'/it OR 'editorial'/it OR 'erratum'/it OR [preprint]/lim OR 'case study'/exp OR 'case report'/exp OR (((case*) NEAR/3 (report* OR stud*)) OR comment):ti

#6 NOT #7 NOT #8

***PICO 9b.***

(P)Adult cardiac surgery with severe bleeding after cardiopulmonary bypass;
(I) fibrinogen

(C）cryoprecipiate; no use

(O) transfusion; bleeding; postoperative complications;

MEDLINE

#1 exp "Cardiac Surgical Procedures"[MeSH] OR exp "Coronary Artery Bypass"[MeSH] OR exp "Heart Valve Prosthesis Implantation"[MeSH] OR exp "Heart Transplantation"[MeSH] OR ("heart surgery"[TIAB] OR "cardiac surgery"[TIAB] OR "heart transplant*"[TIAB] OR "aortic surgery" [TIAB]OR "coronary artery bypass graft*" [TIAB]OR "valve replacement" [TIAB]OR "valve repair"[TIAB] OR "congenital heart surgery"[TIAB]）

#2 exp "Cardiopulmonary bypass"[MeSH] OR exp "Extracorporeal Circulation"[MeSH] OR ("cardiopulmonary bypass"[TIAB] OR "extracorporeal circulation"[TIAB])

#3 exp "hemorrhage"[MeSH] OR exp "blood loss, surgical"[MeSH] OR exp "blood coagulation disorders"[MeSH] OR exp "hemostasis"[MeSH] OR "hemostasis"[TIAB] OR "blood loss"[TIAB] OR "hemorrhage"[TIAB] OR "coagulopathy"[TIAB]

#4 exp "fibrinogen"[MeSH] "fibrinogen"[TIAB] OR "cryoprecipitat*"[TIAB]

#5 #1 AND #2 AND #3 AND #4

#6 #5 AND ("2000"[dp] : "3000"[dp]))

#7 exp "Adolescent"[MeSH] OR exp "Child"[MeSH] OR exp "Infant"[MeSH] OR ("Infant"[TI] OR"Pediatric"[TI] OR"Neonate*"[TI])

#8 "Review"[PT] OR"Editorial"[PT] OR "Comment"[PT] OR "Letter"[PT] OR "News"[PT] OR "Congress"[PT] OR "Published Erratum"[PT] OR"Case Reports"[PT]OR (book*[PT])

#6 NOT #7 NOT #8

Embase

#1 'heart surgery'/exp OR 'cardiovascular surgery'/exp OR 'aortic surgery'/exp OR ('heart surgery':ti,ab OR 'cardiac surgery':ti,ab OR 'heart transplant*':ti,ab OR 'aortic surgery':ti,ab OR 'coronary artery bypass graft*':ti,ab OR 'valve replacement':ti,ab OR 'valve repair':ti,ab OR 'congenital heart surgery':ti,ab)

#2 'cardiopulmonary bypass'/exp OR 'extracorporeal circulation'/exp OR ('cardiopulmonary bypass':ti,ab OR 'extracorporeal circulation':ti,ab)

#3 'bleeding'/exp OR 'hemostasis'/exp OR 'blood loss':ti,ab OR 'hemorrhage':ti,ab OR 'bleeding':ti,ab OR 'coagulopathy':ti,ab

#4 'fibrinogen'/exp OR 'cryoprecipitation'/exp OR 'fibrinogen':ti,ab OR 'cryoprecipitat*':ti,ab

#5 #1 AND #2 AND #3 AND #4

#6 #5 AND [2000-2024]/py

#7 'juvenile'/exp OR ('infant':ti OR 'pediatric':ti OR 'neonate*':ti)

#8 [Conference Abstract]/lim OR 'editorial'/it OR 'letter'/it OR 'note'/it OR 'chapter'/it OR 'conference abstract'/it OR 'conference review'/it OR 'editorial'/it OR 'erratum'/it OR [preprint]/lim OR 'case study'/exp OR 'case report'/exp OR (((case*) NEAR/3 (report* OR stud*)) OR comment):ti

#6 NOT #7 NOT #8

**Question 10: Should intraoperative cell salvage be routinely used to reduce allogeneic transfusion?**

***PICO 10.***

(P)Adult cardiac surgery under cardiopulmonary bypass;
(I) cell salvage

(C）not use

(O) transfusion requirements, Bleeding; postoperative complications;

MEDLINE

#1 exp "Cardiac Surgical Procedures"[MeSH] OR exp "Coronary Artery Bypass"[MeSH] OR exp "Heart Valve Prosthesis Implantation"[MeSH] OR exp "Heart Transplantation"[MeSH] OR ("heart surgery"[TIAB] OR "cardiac surgery"[TIAB] OR "heart transplant*"[TIAB] OR "aortic surgery" [TIAB]OR "coronary artery bypass graft*" [TIAB]OR "valve replacement" [TIAB]OR "valve repair"[TIAB] OR "congenital heart surgery"[TIAB]）

#2 exp "Cardiopulmonary bypass"[MeSH] OR exp "Extracorporeal Circulation"[MeSH] OR ("cardiopulmonary bypass"[TIAB] OR "extracorporeal circulation"[TIAB])

#3 exp "operative blood salvage"[MeSH] OR "cell salvage"[TIAB] OR "cell saver "[TIAB] OR "blood salvage"[TIAB] OR "blood saver"[TIAB]

#4 #1 AND #2 AND #3

#5 #4 AND ("2000"[dp] : "3000"[dp]))

#6 exp "Adolescent"[MeSH] OR exp "Child"[MeSH] OR exp "Infant"[MeSH] OR ("Infant"[TI] OR"Pediatric"[TI] OR"Neonate*"[TI])

#7 "Review"[PT] OR"Editorial"[PT] OR "Comment"[PT] OR "Letter"[PT] OR "News"[PT] OR "Congress"[PT] OR "Published Erratum"[PT] OR"Case Reports"[PT]OR (book*[PT])

#5 NOT #6 NOT #7

Embase

#1 'heart surgery'/exp OR 'cardiovascular surgery'/exp OR 'aortic surgery'/exp OR ('heart surgery':ti,ab OR 'cardiac surgery':ti,ab OR 'heart transplant*':ti,ab OR 'aortic surgery':ti,ab OR 'coronary artery bypass graft*':ti,ab OR 'valve replacement':ti,ab OR 'valve repair':ti,ab OR 'congenital heart surgery':ti,ab)

#2 'cardiopulmonary bypass'/exp OR 'extracorporeal circulation'/exp OR ('cardiopulmonary bypass':ti,ab OR 'extracorporeal circulation':ti,ab)

#3 'blood salvage'/exp OR 'cell salvage':ti,ab OR 'cell saver ':ti,ab OR 'blood salvage':ti,ab OR 'blood saver':ti,ab

#4 #1 AND #2 AND #3

#5 #4 AND [2000-2024]/py

#6 'juvenile'/exp OR ('infant':ti OR 'pediatric':ti OR 'neonate*':ti)

#7 [Conference Abstract]/lim OR 'editorial'/it OR 'letter'/it OR 'note'/it OR 'chapter'/it OR 'conference abstract'/it OR 'conference review'/it OR 'editorial'/it OR 'erratum'/it OR [preprint]/lim OR 'case study'/exp OR 'case report'/exp OR (((case*) NEAR/3 (report* OR stud*)) OR comment):ti

#5 NOT #6 NOT #7

**Question 11: Should autologous platele-rich plasmapheresis (APP) be used before CPB to reduce allogenic transfusion?**

***PICO 11.***

(P)Adult cardiac surgery under cardiopulmonary bypass;
(I) autologous platelet-rich plasmapheresis; autologous platelet separation

(C）not use

(O) transfusion requirements, Bleeding; postoperative complications;

MEDLINE

#1 exp "Cardiac Surgical Procedures"[MeSH] OR exp "Coronary Artery Bypass"[MeSH] OR exp "Heart Valve Prosthesis Implantation"[MeSH] OR exp "Heart Transplantation"[MeSH] OR ("heart surgery"[TIAB] OR "cardiac surgery"[TIAB] OR "heart transplant*"[TIAB] OR "aortic surgery" [TIAB]OR "coronary artery bypass graft*" [TIAB]OR "valve replacement" [TIAB]OR "valve repair"[TIAB] OR "congenital heart surgery"[TIAB]）

#2 exp "Cardiopulmonary bypass"[MeSH] OR exp "Extracorporeal Circulation"[MeSH] OR ("cardiopulmonary bypass"[TIAB] OR "extracorporeal circulation"[TIAB])

#3 "autologous platelet"[TIAB] OR "autologous plasma "[TIAB]

#4 #1 AND #2 AND #3

#5 #4 AND ("2000"[dp] : "3000"[dp]))

#6 exp "Adolescent"[MeSH] OR exp "Child"[MeSH] OR exp "Infant"[MeSH] OR ("Infant"[TI] OR"Pediatric"[TI] OR"Neonate*"[TI])

#7 "Review"[PT] OR"Editorial"[PT] OR "Comment"[PT] OR "Letter"[PT] OR "News"[PT] OR "Congress"[PT] OR "Published Erratum"[PT] OR"Case Reports"[PT]OR (book*[PT])

#5 NOT #6 NOT #7

Embase

#1 'heart surgery'/exp OR 'cardiovascular surgery'/exp OR 'aortic surgery'/exp OR ('heart surgery':ti,ab OR 'cardiac surgery':ti,ab OR 'heart transplant*':ti,ab OR 'aortic surgery':ti,ab OR 'coronary artery bypass graft*':ti,ab OR 'valve replacement':ti,ab OR 'valve repair':ti,ab OR 'congenital heart surgery':ti,ab)

#2 'cardiopulmonary bypass'/exp OR 'extracorporeal circulation'/exp OR ('cardiopulmonary bypass':ti,ab OR 'extracorporeal circulation':ti,ab)

#3 'autologous platelet':ti,ab OR 'autologous plasma ':ti,ab

#4 #1 AND #2 AND #3

#5 #4 AND [2000-2024]/py

#6 'juvenile'/exp OR ('infant':ti OR 'pediatric':ti OR 'neonate*':ti)

#7 [Conference Abstract]/lim OR 'editorial'/it OR 'letter'/it OR 'note'/it OR 'chapter'/it OR 'conference abstract'/it OR 'conference review'/it OR 'editorial'/it OR 'erratum'/it OR [preprint]/lim OR 'case study'/exp OR 'case report'/exp OR (((case*) NEAR/3 (report* OR stud*)) OR comment):ti

#5 NOT #6 NOT #7

**Question 12: Should acute normovolemic hemodilution (ANH) be used to reduce allogenic transfusion?**

***PICO 12.***

(P)Adult cardiac surgery under cardiopulmonary bypass;
(I) acute normovelemic hemodilution

(C）not use

(O) transfusion requirements, Bleeding; postoperative complications;

MEDLINE

#1 exp "Cardiac Surgical Procedures"[MeSH] OR exp "Coronary Artery Bypass"[MeSH] OR exp "Heart Valve Prosthesis Implantation"[MeSH] OR exp "Heart Transplantation"[MeSH] OR ("heart surgery"[TIAB] OR "cardiac surgery"[TIAB] OR "heart transplant*"[TIAB] OR "aortic surgery" [TIAB]OR "coronary artery bypass graft*" [TIAB]OR "valve replacement" [TIAB]OR "valve repair"[TIAB] OR "congenital heart surgery"[TIAB]）

#2 exp "Cardiopulmonary bypass"[MeSH] OR exp "Extracorporeal Circulation"[MeSH] OR ("cardiopulmonary bypass"[TIAB] OR "extracorporeal circulation"[TIAB])

#3 "acute normovelemic hemodilution"[TIAB] OR "ANH"[TIAB]

#4 #1 AND #2 AND #3

#5 #4 AND ("2000"[dp] : "3000"[dp]))

#6 exp "Adolescent"[MeSH] OR exp "Child"[MeSH] OR exp "Infant"[MeSH] OR ("Infant"[TI] OR"Pediatric"[TI] OR"Neonate*"[TI])

#7 "Review"[PT] OR"Editorial"[PT] OR "Comment"[PT] OR "Letter"[PT] OR "News"[PT] OR "Congress"[PT] OR "Published Erratum"[PT] OR"Case Reports"[PT]OR (book*[PT])

#5 NOT #6 NOT #7

Embase

#1 'heart surgery'/exp OR 'cardiovascular surgery'/exp OR 'aortic surgery'/exp OR ('heart surgery':ti,ab OR 'cardiac surgery':ti,ab OR 'heart transplant*':ti,ab OR 'aortic surgery':ti,ab OR 'coronary artery bypass graft*':ti,ab OR 'valve replacement':ti,ab OR 'valve repair':ti,ab OR 'congenital heart surgery':ti,ab)

#2 'cardiopulmonary bypass'/exp OR 'extracorporeal circulation'/exp OR ('cardiopulmonary bypass':ti,ab OR 'extracorporeal circulation':ti,ab)

#3 'acute normovelemic hemodilution':ti,ab OR 'ANH ':ti,ab

#4 #1 AND #2 AND #3

#5 #4 AND [2000-2024]/py

#6 'juvenile'/exp OR ('infant':ti OR 'pediatric':ti OR 'neonate*':ti)

#7 [Conference Abstract]/lim OR 'editorial'/it OR 'letter'/it OR 'note'/it OR 'chapter'/it OR 'conference abstract'/it OR 'conference review'/it OR 'editorial'/it OR 'erratum'/it OR [preprint]/lim OR 'case study'/exp OR 'case report'/exp OR (((case*) NEAR/3 (report* OR stud*)) OR comment):ti

#5 NOT #6 NOT #7

**Question 13: Should residual pump blood be infused back into the patients?**

***PICO 13.***

(P)Adult cardiac surgery under cardiopulmonary bypass;
(I) residual pump blood infusion

(C）not use

(O) transfusion requirements, Bleeding; postoperative complications;

MEDLINE

#1 exp "Cardiac Surgical Procedures"[MeSH] OR exp "Coronary Artery Bypass"[MeSH] OR exp "Heart Valve Prosthesis Implantation"[MeSH] OR exp "Heart Transplantation"[MeSH] OR ("heart surgery"[TIAB] OR "cardiac surgery"[TIAB] OR "heart transplant*"[TIAB] OR "aortic surgery" [TIAB]OR "coronary artery bypass graft*" [TIAB]OR "valve replacement" [TIAB]OR "valve repair"[TIAB] OR "congenital heart surgery"[TIAB]）

#2 exp "Cardiopulmonary bypass"[MeSH] OR exp "Extracorporeal Circulation"[MeSH] OR ("cardiopulmonary bypass"[TIAB] OR "extracorporeal circulation"[TIAB])

#3 "residual blood"[TIAB] OR "pump blood"[TIAB]

#4 #1 AND #2 AND #3

#5 #4 AND ("2000"[dp] : "3000"[dp]))

#6 exp "Adolescent"[MeSH] OR exp "Child"[MeSH] OR exp "Infant"[MeSH] OR ("Infant"[TI] OR"Pediatric"[TI] OR"Neonate*"[TI])

#7 "Review"[PT] OR"Editorial"[PT] OR "Comment"[PT] OR "Letter"[PT] OR "News"[PT] OR "Congress"[PT] OR "Published Erratum"[PT] OR"Case Reports"[PT]OR (book*[PT])

#5 NOT #6 NOT #7

Embase

#1 'heart surgery'/exp OR 'cardiovascular surgery'/exp OR 'aortic surgery'/exp OR ('heart surgery':ti,ab OR 'cardiac surgery':ti,ab OR 'heart transplant*':ti,ab OR 'aortic surgery':ti,ab OR 'coronary artery bypass graft*':ti,ab OR 'valve replacement':ti,ab OR 'valve repair':ti,ab OR 'congenital heart surgery':ti,ab)

#2 'cardiopulmonary bypass'/exp OR 'extracorporeal circulation'/exp OR ('cardiopulmonary bypass':ti,ab OR 'extracorporeal circulation':ti,ab)

#3 'residual blood ':ti,ab OR 'pump blood ':ti,ab

#4 #1 AND #2 AND #3

#5 #4 AND [2000-2024]/py

#6 'juvenile'/exp OR ('infant':ti OR 'pediatric':ti OR 'neonate*':ti)

#7 [Conference Abstract]/lim OR 'editorial'/it OR 'letter'/it OR 'note'/it OR 'chapter'/it OR 'conference abstract'/it OR 'conference review'/it OR 'editorial'/it OR 'erratum'/it OR [preprint]/lim OR 'case study'/exp OR 'case report'/exp OR (((case*) NEAR/3 (report* OR stud*)) OR comment):ti

#5 NOT #6 NOT #7

# Supplementary Material C. Examples for TEG/ROTEM Based Hemostatic Interventions and Transfusion Algorithms

**Supplementary Table 1. An Example for TEG based hemostatic Interventions and Transfusion Algorithms^[1]^**

| TEG Result | Hemostasis State | Recommended Treatment |
| --- | --- | --- |
| Angle |  |  |
| <45° | Low fibrinogen level | 0.06 U/kg cryoprecipitate |
| Reaction time (R value) |  |  |
| < 4 minutes | Enzymatic hypercoagulability | Anticoagulant |
| 11-14 minutes | Low clotting factors | 2 U of FFP |
| >14 minutes | Very low clotting factors | 4 U of FFP |
| Maximal amplitude |  |  |
| 46-54 mm | Low platelet function | 0.3 ug/kg DDAVP |
| 41-45 mm | Very low platelet function | 1 U of platelet pheresis |
| ≤40 mm | Extremely low platelet function | 2 U of platelet pheresis |
| >73 mm | Platelet hypercoagulability | Antiplatelet therapy |

DDAVP = 1-deamino-8-D-arginine vasopressin; FFP=fresh frozen plasma; TEG = thrombelastography.

**Supplementary Table 2. An Example for ROTEM based hemostatic Interventions and Transfusion Algorithm^[2]^**

| EXTEM-MCF parameters | | | |
| --- | --- | --- | --- |
| Clot Firmness | EXTEM-MCF | | |
|  | <35mm | 35-45mm | >45mm |
| FIBTEM-MCF |  |  |  |
| <10mm | Cryo/fibrinogen Platelet 1 U | Cryo/fibrinogen Platelet 1 U | Cryo/fibrinogen |
| ≥10mm | Platelets 1-2 U | Platelets 1U | If bleeding is uncontrolled, consider FFP or PCC based on EXTEM-CT as below or consider platelet transfusion in patients on P2Y12 inhibitors |
|  | Diagnosis | Management |  |
| Prolonged CT Values |  |  |  |
| INTEM-CT/HEPTEM-CT ratio > 1.0 | Residual heparin | Protamine 25-50 mg |  |
| EXTEM-CT＞100s or INTEM-CT＞240s | Low coagulation factors | FFP 10-15 mL/kg or PCC 20 IU/kg |  |
| FIBTEM-A10＜5mm | Very low fibrinogen  (100 mg/dL) | Cryo/fibrinogen as above |  |
| Fibrinolysis Patterns† |  |  |  |
| Fibrinolysis 20 min | Fulminant fibrinolysis | TXA 1-2 g or EACA 5-10 g‡ |  |
| Fibrinolysis 20-40 min | Early fibrinolysis | TXA1gorEACA5g |  |
| Fibrinolysis 40 min | Clot retraction or late fibrinolysis | Usually no treatment is required |  |

Abbreviations: A10, amplitude at 10 minutes after coagulation time; APTEM, modified EXTEM test with aprotinin; Cryo, cryoprecipitate; CT, coagulation time; EACA, -aminocaproic acid; EXTEM, tissue factor reagent; FIBTEM, modified EXTEM test with cytochalasin D; FFP, fresh-frozen plasma; HEPTEM, heparinase plus INTEM reagent; INTEM, ellagic acid reagent; MCF, maximum clot firmness; PCC, prothrombin complex concentrate; TXA, tranexamic acid.

*FIBTEM-A10 at 8 mm may be used as a cutoff instead of FIBTEM-MCF at 10 mm. For fibrinogen replacement, Cryo, 10 U, or plasma-derived fibrinogen concentrate, 2 g, is administered. If FIBTEM-A10 is 5 mm, the dose of Cryo or fibrinogen concentrate is doubled.

†EXTEM and APTEM are repeated after each therapeutic intervention.

‡Antifibrinolytic agents are used only if the risk of bleeding is greater than the risk of thrombosis or worsening of disseminated intravascular coagulation.

**Reference**

1. Redfern RE, Fleming K, March RL, et al. Thrombelastography-Directed Transfusion in Cardiac Surgery: Impact on Postoperative Outcomes. Ann Thorac Surg. 2019. 107(5): 1313-1318.
2. Tanaka KA, Bolliger D, Vadlamudi R, et al. Rotational thromboelastometry (ROTEM)-based coagulation management in cardiac surgery and major trauma. J Cardiothorac Vasc Anesth. 2012. 26(6): 1083-93.

# Supplementary material D. Comparison between 2024 Guidelines on Patient Blood Management for Adult Cardiac Surgery under Cardiopulmonary Bypass in China and other International Guidelines

| **Guidelines** | **Recommendations** |
| --- | --- |
| 2024 China PBM guidelines | **Recommendation 1.** CPB circuit with the reduced priming volume is recommended as an effective measure for patient blood management. (Strong Recommendation, High Certainty) |
| 2021 American PBM guidelines^[1]^ | Reduced priming volume in the CPB circuit reduces hemodilution and is indicated for blood conservation. (Class I, Level B-NR) |
| 2024 EACTS/EACTAIC  PBM Guidelines^[2]^ | Implementation of multiple institutional measures to reduce haemodilution during CPB is recommended to reduce anaemia, transfusion and postoperative bleeding. （Class I, Level B） |
| 2024 China PBM guidelines | **Recommendation 2.** Retrograde autologous priming is suggested to reduce perioperative allogeneic blood transfusion in adult cardiac surgery. (Strong Recommendation, Moderate Certainty) |
| 2019EACTS/EACTA/EBCP CPB Guidelines^[3]^ | Retrograde and antegrade autologous primings are recommended as part of a blood conservation strategy to reduce transfusions.（Class I,Level A） |
| 2021 American PBM guidelines | Retrograde autologous priming of the CPB circuit should be used wherever possible. (Class I, Level B-R) |
| 2024 EACTS/EACTAIC PBM guidelines | Autologous priming, either retrograde or antegrade, is recommended as part of a blood conservation strategy. （Class I,Level A） |
| 2024 China  PBM guidelines | **Recommendation 4**. In patients with preoperative hypoalbuminemia, adding human serum albumin into the priming solution may be considered(Conditional Recommendation, Low Certainty ). |
| 2021 American PBM guidelines | It is reasonable to administer human albumin after cardiac surgery to provide intravascular volume replacement and minimize the need for transfusion. (Class IIA, Level B-R) |
| 2024 China PBM guidelines | **Recommendation 5**. Hydroxyethyl starch solution is not recommended for the priming solution (Strong Recommendation, High Certainty). Gelatin solution may be considered for the priming solution (Conditional Recommendation, Very Low Certainty ). |
| 2019EACTS/EACTA/EBCP CPB Guidelines | The use of modern low-molecular-weight starches in priming and non-priming solutions to reduce bleeding and transfusions is not recommended（Class III,Level C）. |
| 2021 American PBM guidelines | Hydroxyethyl starch is not recommended as a volume expander in cardiopulmonary bypass patients as it may increase the risk of bleeding. (Class III: No Benefit, Level B-R) |
| 2024 EACTS/EACTAIC  PBM Guidelines | Combining the priming volume with colloids to reduce transfusions is not recommended.（Class III，Level:A）  The use of modern low-molecular-weight starches in priming and non-priming solutions to reduce bleeding and transfusions is not recommended.(Class III,Level B) |
| 2024 China PBM guidelines | **Recommendation 6**. For patients with large priming volumes, large blood volumes, or anticipant large residual pump blood volumes, ultrafiltration should be considered as the part of PBM. (Strong Recommendation, Moderate Certainty) |
| 2024 EACTS/EACTAIC  PBM Guidelines | Modified ultrafiltration should be considered as part of a blood conservation strategy. （Class IIa,Level A） |
| 2024 China  PBM guidelines | **Recommendation 7**. In patients with heparin resistance who could not achieve an activated clotting time (ACT) target of 480 s after the administration of unfractionated heparin 600 U/kg, fresh frozen plasma or antithrombin Ⅲ concentrate could be considered (Conditional Recommendation, Moderate Certainty). |
| 2018 STS/SCA/AmSECT Clinical Practice Anticoagulation Guidelines^[4]^ | Heparin is by far the most commonly used anticoagulant during the conduct of cardiac operations, whether done with or without CPB.（Class IIa）。 |
| 2019EACTS/EACTA/EBCP CPB Guidelines | It is recommended that antithrombin concentrate be used instead of FFP to treat antithrombin deficiency to improve heparin sensitivity.（Class I,Level B）If antithrombin concentrate is unavailable, FFP should be considered to treat antithrombin deficiency to improve heparin sensitivity.（Class IIa,Level C）FFP should not be used prophylactically during CPB to reduce perioperative blood loss.（ClassIIII,Level B） |
| 2024 EACTS/EACTAIC  PBM Guidelines | When starting surgery, AT supplementation is recommended in patients with AT deficiency to improve heparin sensitivity. (Class I,Level A) |
| 2024 China PBM guidelines | **Recommendation 8**. A continuous monitoring of heparin residual and rebound may be considered from the initial protamine neutralization to 6h after surgery (Conditional Recommendation, Moderate Certainty). |
| 2018STS/SCA/AmSECT Clinical Practice Anticoagulation Guidelines | Because of the risk of heparin rebound in patients requiring high doses of heparin and with prolonged CPB times, low dose protamine infusion (25 mg/h) for up to 6 hours after the end of CPB may be considered as part of a multimodality blood conservation program. (Class IIb,Level C) |
| 2024 EACTS/EACTAIC  PBM Guidelines | Protamine administration is recommended in a protamineto-heparin dosing ratio d <1.0 to avoid overdosing and reduce bleeding complications.（Class I,Level B）  Individualized heparin and protamine management should be considered to reduce postoperative coagulation abnormalities and bleeding complications after cardiac surgery with CPB.(Class IIa,Level B) |
| 2024 China PBM guidelines | **Recommendation 9**. It is recommended to maintain ACT above 480 seconds (celite method) during CPB (Strong recommendation, Moderate Certainty). It is reasonable to determine the appropriate target ACT values of different instruments for CPB based on the instructions (Conditional recommendation, Low Certainty). |
| 2018STS/SCA/AmSECT Clinical Practice Anticoagulation Guidelines | It is reasonable to maintain activated clotting time above 480 seconds during CPB. However this minimum threshold value is an approximation and may vary based upon the bias of the instrument being used. For instruments using ‘maximal activation’ of whole blood or microcuvette technology, values above 400 seconds are frequently considered therapeutic.（Class IIa ,Level C） |
| 2019EACTS/EACTA/EBCP CPB Guidelines | ACT above 480 s during CPB should be considered in CPB with uncoated equipment and cardiotomy suction. The required target ACT is dependent on the type of equipment used.（Class IIa ,Level C） |
| 2024 EACTS/EACTAIC  PBM Guidelines | Management of heparin anticoagulation with either ACTguided measurements or heparin-level guided measurements is recommended.(Class I,Level A) |
| 2024 China PBM guidelines | **Recommendation 10**. The ACT value should be monitored regularly during CPB (every 30-60 minutes) to guidethe anticoagulation management (Strong recommendation, Low Certainty). |
| 2018STS/SCA/AmSECT Clinical Practice Anticoagulation Guidelines | A functional whole blood test of anticoagulation, in the form of a clotting time, should be measured and should demonstrate adequate anticoagulation before initiation of, and at regular intervals during cardiopulmonary bypass.（Class I,Level C） |
| 2019 EACTS/EACTA/EBCP CPB Guidelines | In the absence of individual heparin dosing tools, it is recommended that ACT tests be performed at regular intervals based on institutional protocols, and heparin doses have to be given accordingly.（Class I,Level C） |
| 2024 China PBM guidelines | **Recommendation 11**. For patients with coagulopathy, thromboelastogram (TEG) / rotational thromboelastometry (ROTEM) is recommended to guide hemostasis management and blood products transfusion after weaning off CPB (Strong recommendation， High Certainty ). |
| 2021 American PBM guidelines | Goal directed transfusion algorithms that incorporate point-of-care testing, such as with viscoelastic devices, are recommended to reduce periprocedural bleeding and transfusion in cardiac surgical patients. (Class I, Level B-R) |
| 2024 China  PBM guidelines | **Recommendation 12**. The prophylactic administration of tranexamic acid is recommended to reduce bleeding and blood product transfusion. (Strong Recommendation,High Certainty). |
| 2021 American PBM guidelines | Use of synthetic antifibrinolytic agents, such as epsilon-aminocaproic acid (EACA) or tranexamic acid, reduces blood loss and blood transfusion during cardiac procedures and is indicated for blood conservation (Class I, Level A). |
| 2024 EACTS/EACTAIC  PBM Guidelines | Antifibrinolytic therapy is recommended to reduce bleeding and transfusions of blood products and reoperations for bleeding.(Class I,Level A) |
| 2024 China PBM guidelines | **Recommendation 13.** A restrictive transfusion strategy is recommended (Strong Recommendation, High Certainty). Generally, it is suggested to maintain a Hemoglobin level (Hb) ≥ 7 g/dL during CPB and maintain Hb ≥ 8 g/dL after CPB (Strong Recommendation, Moderate Certainty). An individualized Hb target strategy based on oxygen delivery-consumption balance may be considered (Conditional Recommendation, Moderate Certainty). |
| 2019EACTS/EACTA/EBCP CPB Guidelines | It is recommended that PRBCs be transfused during CPB if the Hb value is <6.0 g/dl.（Class I,Level C）For HCT values between 18% and 24%, PRBCs may be transfused based on an assessment of the adequacy of tissue oxygenation.（Class IIb,Level B）PRBCs should not be transfused during CPB if the HCT is >24%.（Class IIII,Level C） |
| 2021 American PBM guidelines | On patients undergoing cardiac surgery, a restrictive perioperative allogeneic RBC transfusion strategy is recommended in preference to a liberal transfusion strategy for perioperative blood conservation, as it reduces both transfusion rate and units of allogeneic RBCs without increased risk of mortality or morbidity. (Class I, Level A)  Allogeneic RBC transfusion is unlikely to improve oxygen transport when the hemoglobin concentration is greater than 10 g/dL and is not recommended. (Class III: No Benefit; Level B-R) |
| 2024 EACTS/EACTAIC  PBM Guidelines | Limitation of haemodilution is recommended as part of a blood conservation strategy to reduce bleeding and transfusions.(Class I,Level A)  For HCT values between 18% and 24%, PRBCs may be considered if other measures are not sufficient to maintain the adequacy of tissue oxygenation during CPB, including DO2 and cerebral oximetry.(Class IIb,Level B) |
| 2023 Red Blood Cell Transfusion AABB International Guidelines^[5]^ | The panel recommends that RBC transfusion be administered using a restrictive transfusion strategy of 7 g/dL for most hemodynamically stable adults (strong recommendation, high certainty evidence).  For critically ill children and hospitalized children at risk of critical illness who are hemodynamically stable and without a transfusiondependent hemoglobinopathy, cyanotic cardiac condition, or severe hypoxemia, the international panel recommends a restrictive transfusion strategy in which a transfusion is considered when the hemoglobin level is less than 7 g/dL compared with one of less than 9.5 g/dL (strong recommendation, moderate certainty evidence).  The international panel suggests considering a transfusion threshold for hemodynamically stable children with congenital heartdisease that is based on the cardiac abnormality and stage of surgical repair: 7 g/dL (biventricular repair), 9 g/dL (single-ventricle palliation), or 7 to 9 g/dL (uncorrected congenital heart disease) (conditional recommendation, low certainty evidence). |
| 2024 China  PBM guidelines | **Recommendation 14**. For patients with severe bleeding after CPB due to the confirmed deficiency of clotting factors, prothrombin complex concentrate may be used (Conditional Recommendation， Low Certainty). |
| 2024 EACTS/EACTAIC  PBM Guidelines | In patients with significant bleeding after cardiac surgery due to coagulation factor deficiency, the administration of PCC should be considered instead of FFP to reduce postoperative blood transfusions.(Class IIa,Level B)  In bleeding patients with FXIII activity <70% after CPB, the administration of factor FXIII may be considered to reduce coagulopathy and blood transfusions. (Class IIb,Level B)  The prophylactic use of rFVIIa is not recommended to prevent bleeding complications. (Class III,Level B)  In patients with refractory, non-surgical bleeding, off-label use of rFVIIa may be considered to reduce bleeding complications.(CLass IIb,Level B) |
| 2024 China PBM guidelines | **Recommendation 15.** For patients with severe bleeding and low fibrinogen levels after CPB, fibrinogen supplementation may be considered as it is not inferior to cryoprecipitate in efficacy (Strong Recommendation, Moderate Certainty). |
| 2024 EACTS/EACTAIC  PBM Guidelines | Prophylactic fibrinogen administration is not recommended. (Class III,Level A)  In the bleeding patient with a low fibrinogen level (<1.5 g/L) or the equivalent value in viscoelastic testing, fibrinogen supplementation should be considered to reduce postoperative bleeding and transfusions.(Class IIa,Level B) |
| 2024 China PBM guidelines | **Recommendation 16.** Routine use of ICS is recommended in cardiac surgery (Strong Recommendation, High Certainty). |
| 2019EACTS/EACTA/EBCP CPB Guidelines | Discarding shed blood should be considered.（Class IIa,Level B）  Processing and secondary filtration of red blood cells should be considered to decrease the deleterious effects of reinfused shed blood.（Class IIa，Level B） |
| 2024 EACTS/EACTAIC PBM Guidelines | The use of cell salvage should be considered in order to prevent transfusions. (Class IIa,Level B) |
| 2024 China  PBM guidelines | **Recommendation 18.** ANH is suggested for use in patients with stable conditions who anticipate prolonged cardiopulmonary bypass time or a high risk of bleeding (Strong Recommendation, Moderate Certainty). |
| 2021 American PBM guidelines | Acute normovolemic hemodilution is a reasonable method to reduce bleeding and transfusion. (Class IIA, Level of Evidence A) |
| 2024 EACTS/EACTAIC  PBM Guidelines | A preoperative autologous blood donation in patients with haemoglobin levels over 110 g/L may be considered to reduce postoperative transfusions.（Class IIb，Level B）Acute normovolaemic haemodilution may be considered to reduce postoperative transfusions.(Class IIb,Level A) |
| 2024 China PBM guidelines | **Recommendation 19.** After CPB, the residual pump blood in the circuit should be transfused back into the patients (Strong recommendation, High Certainty). |
| 2019EACTS/EACTA/EBCP CPB Guidelines | Retransfusion of the residual volume of the CPB circuit at the end of the procedure is recommended as a part of a blood management programme to minimize allogeneic blood transfusions.（Class I,Level C）  Retransfusion of the processed residual volume of the CPB circuit at the end of the procedure should be considered for minimizing the risks of allogeneic blood transfusions.（ClassIIa,Level B） |

**Reference**

1. Tibi P, McClure RS, Huang J, et al. STS/SCA/AmSECT/SABM Update to the Clinical Practice Guidelines on Patient Blood Management. Ann Thorac Surg. 2021;112(3):981-1004.
2. Casselman FPA, Lance MD, Ahmed A, et al. 2024 EACTS/EACTAIC Guidelines on patient blood management in adult cardiac surgery in collaboration with EBCP. Eur J Cardiothorac Surg. 2024 Oct 10:ezae352.
3. Authors/Task Force Members; Kunst G, Milojevic M, et al. 2019 EACTS/EACTA/EBCP guidelines on cardiopulmonary bypass in adult cardiac surgery. Br J Anaesth. 2019;123(6):713-757.
4. Shore-Lesserson L, Baker RA, Ferraris V, et al. STS/SCA/AmSECT Clinical Practice Guidelines: Anticoagulation during Cardiopulmonary Bypass. J Extra Corpor Technol. 2018;50(1):5-18.
5. Carson JL, Stanworth SJ, Guyatt G, et al. Red Blood Cell Transfusion: 2023 AABB International Guidelines. JAMA. 2023;330(19):1892-1902.
